# Metformin Exposure in Pregnancy and Fetal Programming: A Focused Review of Contemporary Evidence

**DOI:** 10.3390/biomedicines14081867

**Published:** 2026-08-20

**Authors:** Miroslava Gojnic Dugalic, Stefan Dugalic, Milos Milincic, Katarina Ivanovic

**Affiliations:** 1 Clinic for Gynecology and Obstetrics, University Clinical Centre of Serbia, 11000 Belgrade, Serbia; miroslavagojnicdugalic@yahoo.com (M.G.D.); stef.dugalic@gmail.com (S.D.); milosmilincic@gmail.com (M.M.); 2 Faculty of Medicine, University of Belgrade, 11000 Belgrade, Serbia

**Keywords:** fetal programming, DOHaD, metformin, pregnancy, gestational diabetes, type 2 diabetes, polycystic ovary syndrome, placenta, offspring outcomes, women’s health

## Abstract

**Background:** Fetal programming, conceptualized within the Developmental Origins of Health and Disease framework, describes how intrauterine exposures may shape long-term offspring physiology and disease susceptibility. Metformin is increasingly used during pregnancy for gestational diabetes mellitus, type 2 diabetes mellitus, and selected cases of polycystic ovary syndrome. Its developmental interpretation is complex because the drug improves the maternal metabolic environment but also crosses the placenta and directly exposes the fetus. **Methods:** This focused narrative review used iterative, targeted searches of PubMed/MEDLINE, Scopus, Web of Science, and Google Scholar to identify pharmacological, placental, mechanistic, clinical, guideline-based, and offspring follow-up evidence. The literature search covered publications available through 31 May 2026. The manuscript was subsequently revised during the editorial submission and peer-review process, and the evidence synthesis was re-evaluated against this final literature cut-off date. Earlier landmark studies were retained where necessary for historical, pharmacological, or methodological context. **Results:** Metformin reduces hepatic glucose production and maternal insulin resistance and may limit gestational weight gain, insulin requirements, neonatal hypoglycemia, and excessive fetal growth in selected pregnancies. Placental transfer creates biological plausibility for direct effects on AMPK, mitochondrial, mTOR, nutrient-sensing, and epigenetic pathways. Long-term studies are broadly reassuring regarding neurodevelopment and major metabolic disease, but findings on growth trajectory, adiposity, and small-for-gestational-age risk remain heterogeneous and indication-dependent. **Conclusions:** Metformin should be interpreted neither as uniformly beneficial nor as developmentally neutral. Its use should be individualized according to maternal phenotype, indication, glycemic benefit, placental function, fetal growth, dose, timing, and the limitations of long-term offspring evidence.

## 1. Introduction

Pregnancy is a period of exceptional biological plasticity. The fetus continuously responds to maternal nutritional, endocrine, metabolic, inflammatory, vascular, and pharmacological signals. These signals influence immediate growth and organ maturation, but they may also alter physiological set-points and disease susceptibility across the life course. The fetal origins hypothesis has therefore evolved into the broader Developmental Origins of Health and Disease (DOHaD) framework, in which the intrauterine environment is understood as a determinant of later metabolic, cardiovascular, immune, neurodevelopmental, and reproductive health [1,2,3].

In pregnancies complicated by hyperglycemia, obesity, or insulin resistance, developmental programming is driven by more than glucose alone. Maternal dyslipidemia, inflammation, oxidative stress, altered placental nutrient transport, and fetal hyperinsulinemia may influence adipocyte differentiation, pancreatic beta-cell function, hepatic metabolism, vascular development, and appetite regulation. Epigenetic mechanisms and placental adaptation provide plausible links between these exposures and persistent offspring phenotypes [4,5,6].

Metformin is a biguanide insulin-sensitizing agent used widely outside pregnancy and increasingly during pregnancy. It decreases hepatic glucose production, improves peripheral insulin sensitivity, and influences cellular energy balance through mechanisms involving mitochondrial function, AMP-activated protein kinase (AMPK), nutrient sensing, inflammatory pathways, and the gut-metabolic axis [7]. In pregnancy, it is used principally for gestational diabetes mellitus (GDM), as an adjunct to insulin in type 2 diabetes mellitus (T2DM), and in selected women with polycystic ovary syndrome (PCOS).

Metformin differs fundamentally from insulin in fetal exposure. Insulin does not cross the placenta in clinically meaningful amounts, whereas metformin is actively transported across the placenta and fetal concentrations may approach maternal concentrations [5,8]. This creates a dual developmental model. Metformin may indirectly protect the fetus by improving maternal glycemia and reducing excessive nutrient exposure, while simultaneously exerting direct effects on placental and fetal energy-sensing pathways.

Short-term evidence is generally reassuring, and several trials report lower maternal weight gain, reduced insulin requirements, less neonatal hypoglycemia, or lower birthweight compared with insulin-based strategies. However, reduced fetal overgrowth is not synonymous with developmental neutrality. Signals related to small-for-gestational-age birth, accelerated postnatal growth, childhood adiposity, and phenotype-specific effects have generated ongoing uncertainty. In this review, developmental neutrality refers to the absence of a persistent exposure-related effect on offspring growth, body composition, metabolic, neurodevelopmental, immune, reproductive, or cardiovascular outcomes.

The aim of this focused review is to synthesize contemporary evidence on metformin exposure during pregnancy through the lens of fetal programming. Several recent reviews have summarized the clinical use of metformin during pregnancy, with particular emphasis on short-term maternal, fetal, and neonatal outcomes, while others have focused primarily on long-term offspring growth and cardiometabolic health [9,10]. The distinctive contribution of the present review is therefore not to provide another general assessment of whether metformin is uniformly safe or effective. Instead, it applies the Developmental Origins of Health and Disease framework to integrate four related but methodologically distinct evidence streams: maternal metabolic effects; metformin pharmacokinetics and placental transfer; experimental placental, fetal, and cellular mechanisms; and longitudinal human offspring outcomes.

This approach additionally treats GDM, pregestational T2DM, PCOS, and maternal obesity without diabetes as biologically and clinically distinct exposure contexts. It integrates the effects of maternal disease severity, confounding by indication, dose, gestational timing, renal clearance, placental transporter activity, fetal sex, supplemental insulin, and the postnatal environment. The principal novelty of this synthesis therefore lies in linking mechanistic plausibility with the strength and limitations of human clinical evidence and translating these findings into an indication-specific framework for clinical interpretation, monitoring, and shared decision-making. Particular emphasis is placed on placental transfer, cellular and epigenetic pathways, differences among GDM, T2DM, PCOS, and maternal obesity, and long-term offspring growth, metabolic, neurodevelopmental, immune, and reproductive outcomes.

## 2. Materials and Methods

### 2.1. Study Design

This article was designed as a focused narrative review because the research question spans heterogeneous evidence domains, including pharmacology, placental biology, experimental and epigenetic mechanisms, randomized and observational clinical studies, clinical guidelines, and long-term offspring follow-up. The objective was to identify, organize, critically interpret, and integrate contemporary evidence on metformin exposure during pregnancy and its potential relevance to fetal development and long-term offspring health. In accordance with established principles for narrative reviews, the methodology emphasized a clearly defined scope, transparent reporting of the literature sources, thematic prioritization of evidence, and qualitative synthesis rather than exhaustive study retrieval or quantitative pooling [11]. Accordingly, this review was not intended to provide a systematic or exhaustive synthesis, generate pooled treatment estimates, or replace a formal systematic review or meta-analysis. Methodological reporting was additionally informed by the SANRA domains, including justification of the review question, description of the literature search, appropriate referencing, presentation of the level of evidence, and reporting of clinically relevant endpoint data [12].

The review was structured around the principle that treatment during pregnancy should be evaluated in relation to both immediate maternal and neonatal benefits and potential long-term developmental consequences. Particular attention was given to the interaction among maternal metabolic phenotype, placental function, direct fetal drug exposure, fetal growth, and the postnatal environment.

### 2.2. Literature Sources and Search Strategy

A targeted and iterative literature search was conducted in PubMed/MEDLINE, Scopus, Web of Science, and Google Scholar. The literature search covered publications available through 31 May 2026, which was defined as the final literature cut-off date for the present review. The manuscript subsequently underwent substantial revision during the editorial submission and peer-review process; during this period, the selected evidence and narrative synthesis were re-evaluated to ensure consistency with the literature available up to this cut-off date. Accordingly, the review focused primarily on English-language publications issued between January 2015 and 31 May 2026, supplemented by earlier landmark studies where required for historical, pharmacological, or methodological context. No publication released after the stated literature cut-off date was incorporated into the evidence synthesis. Earlier landmark publications were additionally retained when they were considered essential for explaining the Developmental Origins of Health and Disease framework, metformin pharmacology and placental transfer, major randomized trials, or established offspring follow-up cohorts.

The search strategy combined terms related to the exposure, maternal indication, biological mechanism, and offspring outcome. The principal Boolean structure was: (“metformin” OR “biguanide”) AND (“pregnancy” OR “gestational diabetes mellitus” OR “type 2 diabetes mellitus” OR “polycystic ovary syndrome” OR “maternal obesity”) AND (“placental transfer” OR “fetal exposure” OR “pharmacokinetics” OR “fetal programming” OR “developmental origins of health and disease” OR “DOHaD” OR “AMPK” OR “mTOR” OR “mitochondria” OR “epigenetics” OR “birthweight” OR “small for gestational age” OR “offspring growth” OR “adiposity” OR “cardiometabolic outcomes” OR “neurodevelopment” OR “immune outcomes” OR “long-term follow-up”). Search syntax and combinations were adapted to the indexing requirements of each database and were refined iteratively according to the thematic domain being examined.

**Figure 1:**
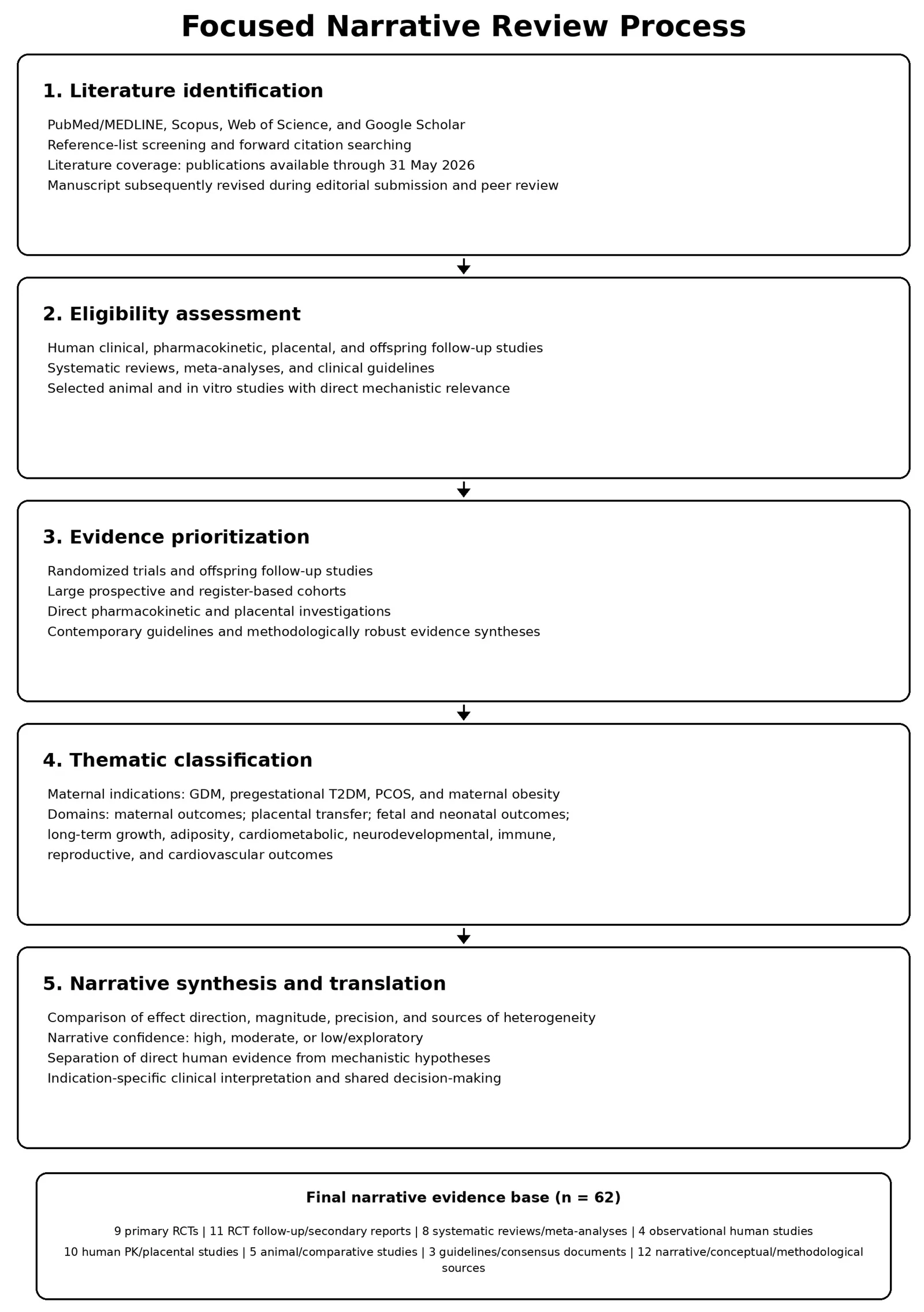
Structured workflow for the literature identification, evidence selection, prioritization, thematic classification, and narrative synthesis. The figure summarizes the targeted and iterative search strategy used for this focused narrative review. The literature search covered publications available through 31 May 2026, which was defined as the final literature cut-off date for the review. The manuscript was subsequently revised during the editorial submission and peer-review process, with the evidence synthesis re-evaluated against this final literature cut-off date. Human clinical evidence was prioritized, whereas experimental evidence was included when it contributed directly to the interpretation of placental biology, fetal development, or developmental mechanisms. The final narrative evidence base comprised 62 publications, and their distribution by primary study-design category is shown in the final panel. Because record-level identification, deduplication, screening, exclusion, and inclusion counts were not prospectively collected, the figure should not be interpreted as a PRISMA 2020 study-selection diagram. Evidence was organized according to maternal indication and outcome domain and interpreted using narrative confidence descriptors rather than formal outcome-specific GRADE ratings. Abbreviations: GDM, gestational diabetes mellitus; PCOS, polycystic ovary syndrome; T2DM, type 2 diabetes mellitus.

Searches were supplemented by screening the reference lists of major randomized trials, systematic reviews, meta-analyses, clinical guidelines, narrative reviews, and relevant offspring follow-up publications. Forward citation searching was also used to identify later reports originating from major pregnancy trials and established maternal–offspring cohorts. Because the purpose of the search was to support a focused narrative synthesis rather than an exhaustive systematic review, study identification proceeded according to relevance to the predefined thematic domains and continued until additional searches did not identify materially new clinical evidence or major conceptual themes. Because the search was iterative and designed to support a focused narrative synthesis, formal PRISMA-style record counts were not prospectively recorded and were therefore not retrospectively reconstructed. The literature identification, selection, prioritization, and synthesis process is summarized schematically in Figure 1. Accordingly, Figure 1 should be interpreted as a structured narrative-review workflow rather than as a PRISMA 2020 study-selection diagram.

The final narrative synthesis included 62 publications. For descriptive purposes, each publication was assigned to one primary evidence category according to its principal study design or role in the synthesis. The evidence base therefore comprised 9 primary randomized controlled trials; 11 randomized-trial follow-up studies, secondary analyses, or randomized substudies; 8 systematic reviews or meta-analyses; 4 register-based or other observational human studies; 10 human pharmacokinetic or placental mechanistic studies; 5 animal or comparative experimental studies; 3 clinical guidelines or consensus documents; and 12 narrative, conceptual, or methodological sources. These counts refer to publications rather than unique parent trials or cohorts, because several major randomized studies generated separate offspring follow-up reports or secondary analyses. They are intended to characterize the composition and breadth of the evidence base rather than to represent a PRISMA-style screening denominator. Record-level counts for database identification, deduplication, screening, and exclusion were not prospectively collected and were therefore not retrospectively reconstructed. The study-level characteristics of all 62 publications are presented in Supplementary Table S1, while their distribution by primary evidence category is summarized schematically in Figure 1.

### 2.3. Guideline and Regulatory Framework

Contemporary clinical guidance was reviewed to contextualize the position of metformin across different maternal indications. Particular consideration was given to the American Diabetes Association Standards of Care in Diabetes—2026 and the 2025 joint clinical practice guideline of the Endocrine Society and the European Society of Endocrinology [13,14]. Guideline recommendations were interpreted separately from primary clinical and mechanistic evidence and were not used as substitutes for critical evaluation of the underlying studies.

The review focused on whether insulin remained the preferred pharmacological treatment, the circumstances in which metformin could be considered, the need for supplemental insulin, and clinical situations requiring additional caution. These included suspected placental insufficiency, established or increased risk of fetal growth restriction, chronic hypertension, diabetic nephropathy, impaired maternal renal function, and inadequate glycemic response. The joint Endocrine Society and European Society of Endocrinology guideline was particularly relevant because it advised against the routine addition of metformin to insulin in pregnancies complicated by T2DM, considering the balance between reduced large-for-gestational-age birth and the potential risk of small-for-gestational-age birth and altered childhood body composition [14].

Differences among guideline recommendations were reported rather than harmonized into a single universal position, because they may reflect differences in the populations considered, healthcare resources, regulatory status, interpretation of placental transfer, and certainty assigned to long-term offspring evidence. Regulatory approval and product labeling were considered separately from professional clinical recommendations, as regulatory status varies among jurisdictions and does not necessarily indicate uniform clinical practice.

### 2.4. Eligibility Criteria

Eligible publications included peer-reviewed randomized controlled trials, prospective and retrospective cohort studies, register-based studies, pharmacokinetic investigations, placental studies, long-term offspring follow-up studies, systematic reviews, meta-analyses, and clinical guidance documents. Publications were considered eligible when they examined metformin exposure before conception, during pregnancy, or during lactation and addressed at least one predefined domain: maternal metabolic or obstetric outcomes; placental transfer or function; fetal growth and development; neonatal outcomes; postnatal growth or body composition; cardiometabolic, neurodevelopmental, immune, reproductive, or cardiovascular outcomes in the offspring; or breastfeeding-related infant exposure.

Human evidence was prioritized. Experimental animal and in vitro studies were considered only when they provided mechanistic information directly relevant to human placental biology, fetal development, mitochondrial function, AMPK–mTOR signaling, nutrient sensing, transporter activity, or epigenetic regulation. Systematic reviews and meta-analyses were used to contextualize the consistency and magnitude of clinical findings, whereas clinical guidelines were used to describe the contemporary clinical positioning of metformin rather than as substitutes for primary evidence.

Full-text English-language publications were preferentially included. Conference abstracts were considered only when a corresponding full-text publication was unavailable, the abstract provided sufficient information on the study population, metformin exposure, comparator, and principal outcomes and the findings were relevant to a recently completed or ongoing major clinical study. Findings available only in abstract form were treated as preliminary and were not used as the sole basis for clinical conclusions.

### 2.5. Exclusion Criteria

Publications were excluded when they were unrelated to pregnancy, lactation, placental or fetal development, neonatal outcomes, or health outcomes in exposed offspring. Studies conducted exclusively in non-pregnant populations were excluded unless they provided pharmacological or mechanistic information directly applicable to metformin exposure during pregnancy. Experimental animal and in vitro studies without a clear connection to human placental function, fetal development, or developmental programming were also excluded.

Conference abstracts were excluded when they did not provide sufficient information on the study population, exposure, comparator, methods, or outcomes, or when a corresponding full-text publication was available. Case reports and small case series were not included in the clinical outcome synthesis unless they contributed unique pharmacokinetic or safety information that was unavailable from larger studies. Editorials, commentaries, and expert opinions were used only to contextualize clinical controversies and were not treated as primary evidence of treatment effects.

Potentially overlapping publications were assessed according to the study population, recruitment period, maternal indication, intervention, comparator, offspring age at assessment, and reported outcomes. When multiple publications reported the same outcome from the same trial or cohort at the same follow-up time point, the most complete and methodologically informative report was retained. Separate publications from the same parent study were retained when they reported distinct outcomes, additional follow-up periods, or new offspring assessments.

### 2.6. Evidence Selection, Prioritization, and Narrative Confidence Assessment

Two authors participated in the literature selection and thematic classification. The initial literature search and screening were conducted by one author, while the selected publications, their eligibility, and their allocation to the predefined thematic domains were reviewed by a second author. Any differences regarding study relevance, classification, or interpretation were resolved through discussion and reassessment of the full-text publication.

Because this was a focused narrative review, publications were selected according to their relevance to the predefined thematic domains rather than through exhaustive systematic inclusion. Priority was assigned to randomized controlled trials and their offspring follow-up studies, systematic reviews and meta-analyses, large prospective or register-based cohorts, contemporary clinical guidelines, and pharmacokinetic studies directly examining maternal, placental, fetal, or breast-milk exposure. Evidence from gestational diabetes mellitus, pregestational type 2 diabetes mellitus, polycystic ovary syndrome, and maternal obesity was interpreted separately whenever possible because the underlying maternal metabolic phenotype, timing of exposure, treatment comparator, and baseline pregnancy risk differed substantially among these indications.

When several systematic reviews or meta-analyses addressed the same clinical question, priority was given to the most recent methodologically comprehensive synthesis that reported explicit eligibility criteria, study-level characteristics, risk-of-bias or certainty assessments, heterogeneity, and indication-specific results. Earlier reviews were retained only when they included landmark trials, distinct outcomes, longer follow-up periods, or analyses not covered by the more recent synthesis.

Major randomized trials were examined directly rather than represented solely through secondary reviews, particularly when treatment timing, comparator treatment, supplemental insulin use, outcome definitions, absolute event rates, or offspring follow-up required verification. When several publications originated from the same trial or cohort, the primary trial report was used for prespecified maternal, fetal, and neonatal outcomes, while separate follow-up publications were retained only when they reported a distinct offspring age, additional follow-up period, or non-overlapping outcome domain.

To improve transparency in evidence interpretation, each principal body of evidence was assigned a narrative confidence descriptor of high, moderate, or low/exploratory. This domain-level assessment was informed by the principal GRADE considerations of risk of bias, inconsistency, indirectness, imprecision, and potential publication bias [15]. Additional consideration was given to the magnitude and precision of reported effects, evidence of a dose–response relationship, the likelihood and direction of residual confounding, consistency across independent populations, and the applicability of the evidence to the maternal indication and clinical outcome under consideration.

High narrative confidence was assigned when a finding was supported by consistent results from multiple well-conducted randomized trials, robust systematic reviews or meta-analyses, or large complementary human studies with clinically relevant and reasonably precise effect estimates. Moderate confidence was assigned when evidence was derived from a limited number of trials or generally consistent observational studies but remained affected by some combination of heterogeneity, imprecision, incomplete follow-up, indirectness, or residual confounding. Low or exploratory confidence was assigned when conclusions were based primarily on small cohorts, secondary or post hoc analyses, inconsistent observational evidence, substantial attrition, animal experiments, in vitro studies, mechanistic hypotheses, or evidence with limited direct applicability to human pregnancy.

When an included systematic review or meta-analysis reported a formal risk-of-bias or GRADE assessment, those judgments were considered during interpretation. However, because the present article was designed as a focused narrative review rather than an exhaustive systematic review, de novo validated risk-of-bias assessments were not performed for every individual publication, and outcome-specific GRADE evidence profiles were not constructed. The descriptors used in this review should therefore be interpreted as a structured, GRADE-informed approach to narrative communication rather than as formal GRADE certainty ratings.

When findings were inconsistent, studies were compared according to maternal indication and disease severity, comparator treatment, metformin dose and gestational timing, use of supplemental insulin, baseline vascular or renal disease, geographic and ethnic setting, offspring age at assessment, attrition, outcome definition, and measurement method. Greater interpretive weight was given to randomized human evidence, prospectively defined outcomes, adjusted effect estimates, and findings replicated across independent populations. Experimental and mechanistic evidence was used to assess biological plausibility and generate hypotheses but was not considered proof of a clinical effect.

### 2.7. Data Extraction and Thematic Organization

Data from the selected publications were extracted by one author and verified by a second author. For each study, the following information was recorded where available: publication year, study design, geographic setting, sample size, maternal indication for metformin use, baseline maternal metabolic and clinical characteristics, gestational timing of exposure, metformin dose and duration, comparator treatment, use of supplemental insulin, duration of offspring follow-up, attrition, outcome definitions, measurement methods, and adjustment for potential confounders. For clinical studies, numerical effect estimates, measures of variability, and 95% confidence intervals were recorded when reported in the original publication. Any differences in data interpretation or thematic allocation were resolved through discussion and re-examination of the full-text article.

Extracted evidence was initially grouped according to maternal indication: gestational diabetes mellitus, pregestational type 2 diabetes mellitus, polycystic ovary syndrome, and maternal overweight or obesity. Within each maternal indication, evidence was subsequently organized according to the following predefined domains: maternal metabolic and obstetric outcomes; metformin pharmacokinetics and placental transfer; placental and fetal mechanisms; fetal growth and neonatal outcomes; postnatal growth and body composition; cardiometabolic outcomes; neurodevelopmental, immune, reproductive, and cardiovascular outcomes; breastfeeding exposure; and implications for clinical practice and future research.

Experimental evidence was classified separately according to whether it originated from animal studies, human placental tissue, isolated placental cells, or other in vitro models. Mechanistic findings were linked to the corresponding clinical domain only when a biologically plausible relationship could be identified. Experimental findings were not combined with human clinical outcomes or interpreted as direct evidence of clinical benefit or harm.

### 2.8. Qualitative Synthesis

A structured qualitative synthesis was undertaken in sequential stages. First, findings were grouped according to maternal indication, including gestational diabetes mellitus, pregestational type 2 diabetes mellitus, polycystic ovary syndrome, and maternal overweight or obesity. Within each indication, studies were organized according to the predefined clinical, pharmacological, placental, mechanistic, and offspring outcome domains. Findings were then compared according to study design, comparator treatment, gestational timing and duration of metformin exposure, use of supplemental insulin, and duration of offspring follow-up.

For clinical outcomes, the direction, magnitude, and consistency of reported associations or treatment effects were examined. Numerical effect estimates, absolute event rates, and 95% confidence intervals were reported when available and clinically informative. Greater emphasis was placed on the magnitude and precision of the observed effect than on statistical significance alone. Findings were considered convergent when studies with comparable populations, exposure definitions, comparators, and outcome measures reported results in the same direction.

When findings differed among studies, potential sources of heterogeneity were examined, including maternal indication and disease severity, baseline glycemic control, body mass index, chronic hypertension or nephropathy, metformin dose, gestational timing of treatment initiation, supplemental insulin use, comparator treatment, ethnicity and geographic setting, offspring age at assessment, duration of follow-up, attrition, outcome definition, measurement method, and adjustment for maternal and postnatal confounders. Conflicting findings were presented explicitly rather than combined into a single generalized conclusion.

Human randomized and observational evidence, pharmacokinetic studies, placental investigations, animal experiments, and in vitro findings were synthesized as separate evidence streams. Mechanistic findings were labeled according to their source and were used to assess biological plausibility and generate hypotheses. Associations observed in experimental studies or observational human cohorts were not interpreted as proof of a metformin-specific causal effect. In particular, proposed mitochondrial, AMPK–mTOR, transporter-related, and epigenetic mechanisms were presented as biologically plausible pathways unless supported by direct and reproducible human evidence.

No quantitative pooling was performed because of substantial clinical and methodological heterogeneity across maternal indications, treatment comparators, exposure timing, supplemental therapy, offspring age, and outcome definitions. Conclusions were therefore based on qualitative triangulation of the direction and consistency of findings, the methodological strength of the contributing evidence, the narrative confidence descriptors defined in Section 2.6, and the presence of plausible alternative explanations, including confounding by indication. Accordingly, the qualitative synthesis was based on 62 publications spanning randomized, observational, pharmacokinetic, placental, experimental, evidence-synthesis, guideline, and methodological sources, with the complete study-level evidence map provided in Supplementary Table S1.

### 2.9. Public Health and Translational Perspective

The evidence was additionally interpreted through a life-course, public health, and clinical implementation perspective. Pregnancy was considered both a therapeutic window for improving maternal metabolic health and a developmental window during which maternal disease, placental function, fetal nutrient exposure, pharmacological treatment, and the postnatal environment may jointly influence future health. Consequently, short-term maternal and neonatal outcomes were considered alongside potential longer-term effects on offspring growth, adiposity, metabolic health, neurodevelopment, and cardiovascular risk.

The translational assessment considered not only clinical efficacy and safety outcomes but also treatment feasibility, route of administration, gastrointestinal tolerability, treatment adherence, need for supplemental insulin, maternal preferences, healthcare-resource requirements, and differences in regulatory and guideline positions. Particular attention was given to clinical circumstances in which the expected maternal benefit might coexist with uncertainty concerning fetal growth or long-term offspring outcomes.

The purpose of this perspective was not to classify metformin as universally safe or unsafe or to formulate recommendations that were independent of maternal indication. Instead, the evidence was interpreted to support individualized benefit–risk assessment and shared decision-making according to maternal metabolic phenotype, disease severity, glycemic response, vascular and renal comorbidity, placental function, fetal growth, available treatment alternatives, and patient values. Areas in which evidence remained limited or inconsistent were explicitly identified as priorities for long-term follow-up and future research. Because long-term offspring effects cannot currently be quantified with precision, this uncertainty should be disclosed explicitly as an ethical component of informed consent and documented as part of the shared decision-making process.

## 3. Results and Thematic Synthesis

### 3.1. Pharmacological Characteristics, Placental Transfer, and Fetal Exposure

Metformin is a hydrophilic, positively charged biguanide that undergoes minimal clinically relevant metabolism and is eliminated predominantly unchanged by the kidneys. Its glucose-lowering effect is mediated primarily through reduced hepatic glucose production and improved insulin sensitivity, although mitochondrial respiratory activity, AMPK-related signaling, glucagon pathways, lipid metabolism, inflammation, and gut-derived signals may also contribute [7]. These pathways are relevant to pregnancy because they also participate in placental energy regulation, nutrient sensing, cellular proliferation, and fetal growth.

The administered dose and the timing of treatment vary among maternal indications and clinical trials. In studies of GDM, metformin has commonly been initiated at 500 mg once daily and progressively titrated according to gastrointestinal tolerance and glycemic response, with maximum daily doses generally ranging from 2000 to 2500 mg. In trials involving pregestational T2DM, metformin has frequently been administered at 1000 mg twice daily as an adjunct to insulin. Gradual dose escalation is clinically important because gastrointestinal intolerance may limit the achieved dose and treatment adherence. The prescribed dose should therefore not be assumed to represent the actual maternal or fetal exposure, particularly when adherence, treatment duration, renal function, and supplemental insulin are not reported.

Pregnancy substantially alters metformin pharmacokinetics. In a longitudinal pharmacokinetic study, mean metformin renal clearance increased to 723 ± 243 mL/min in mid-pregnancy and 625 ± 130 mL/min in late pregnancy, compared with 477 ± 132 mL/min postpartum [8]. These changes reflected increases in both glomerular filtration and active tubular secretion. A subsequent study similarly reported increased apparent oral clearance, renal clearance, and volume of distribution during pregnancy. Among women receiving a 500 mg dose, apparent oral clearance was approximately 27% higher and weight-adjusted renal secretion clearance approximately 64% higher during pregnancy than in non-pregnant participants [16]. Consequently, the same oral dose may produce lower or more variable maternal plasma exposure during pregnancy, particularly at lower doses.

Maternal renal function is therefore a major determinant of systemic exposure. Increased glomerular filtration during an uncomplicated pregnancy may lower maternal metformin concentrations, whereas impaired renal function may reduce elimination and increase drug exposure. Renal function should be evaluated before treatment and reassessed when clinically indicated, particularly in women with diabetic nephropathy, hypertensive disease, dehydration, acute illness, or other conditions that may alter renal clearance. However, pregnancy-specific evidence directly relating maternal renal impairment to quantitative fetal metformin exposure remains limited.

Metformin readily reaches the fetal circulation. Earlier human studies reported cord plasma concentrations ranging from undetectable levels to concentrations approaching those in maternal plasma [8]. In a later pharmacokinetic study, the mean venous umbilical cord-to-maternal plasma concentration ratio was 1.4 ± 0.5, while the arterial cord-to-maternal ratio was 1.5 ± 0.5 [16]. These values demonstrate substantial fetal exposure at delivery, but they should not be interpreted as fixed transfer ratios. Cord concentrations depend on the maternal dose, interval between the last dose and delivery, maternal renal clearance, gestational age, placental function, adherence, and the timing of maternal and cord sampling.

The mechanisms governing placental transfer remain incompletely defined and should be distinguished from the transport processes determining maternal systemic exposure. Metformin is a hydrophilic organic cation that depends on membrane transporters for cellular uptake and elimination. In maternal tissues, organic cation transporter 1 (OCT1/SLC22A1) contributes predominantly to hepatic uptake, whereas OCT2/SLC22A2, multidrug and toxin extrusion protein 1 (MATE1/SLC47A1), and MATE2-K/SLC47A2 participate in renal tubular secretion. Pregnancy increases both glomerular filtration and net tubular secretion, resulting in higher metformin renal clearance and, particularly at lower doses, lower maternal systemic exposure [8,16,17]. Consequently, maternal renal function and the activity of renal OCT2 and MATE transporters may influence the amount of drug available for placental transfer, although pregnancy-specific pharmacogenetic data remain insufficient for individualized dose adjustment.

Among placental organic cation transporters, OCT3/SLC22A3 has the strongest evidence for a role in fetal disposition. In the human term placenta, OCT3 is localized predominantly to the basal, fetal-facing membrane of the syncytiotrophoblast and is also detectable in fetal capillaries [18]. In pregnant mice, deletion of Oct3 reduced overall fetal metformin exposure by approximately 47%, while producing only a modest change in maternal systemic exposure, supporting a role in movement of metformin from placental tissue toward the fetal circulation [18]. Human placental proteomic data further indicate that OCT3 abundance increases approximately twofold from the first trimester to term, suggesting that transporter-mediated fetal disposition may vary according to gestational age [19]. However, the mouse knockout findings remain mechanistic evidence and do not quantify the contribution of OCT3 to fetal exposure in individual human pregnancies.

OCT1 and OCT2 are less consistently or less abundantly demonstrated in the human placenta than OCT3. Experimental studies using trophoblast cell lines and animal models suggest that OCT1, OCT2, and other uptake transporters may contribute to placental metformin handling, but their functional importance in the intact human placenta has not been established. OCT1 may be more relevant after the drug has entered the fetal circulation because it is expressed in fetal tissues, including the liver, where it may facilitate tissue-specific uptake and cellular effects. These observations should not be interpreted as evidence that placental OCT1 or OCT2 independently determines human fetal exposure.

The role of placental MATE transporters is even more species-dependent. MATE1 and MATE2-K are important for maternal renal elimination of metformin, but their expression in human placental villous tissue is low, variable, or below reliable quantitative detection in several studies. By contrast, functional placental MATE1 is strongly expressed in rats and contributes to transport of metformin toward the maternal circulation. Recent comparative evidence indicates that rat-specific placental MATE1 expression may partly explain the substantially lower fetal-to-maternal metformin concentration ratio in rats than in humans [20]. Rat developmental studies may therefore underestimate human fetal exposure and should not be extrapolated without consideration of species-specific placental transporter expression.

Placental metformin transfer is unlikely to depend on a single transporter. Human placental perfusion and computational modeling support bidirectional transfer involving both paracellular diffusion and OCT3-mediated transport at the basal membrane [21]. The relative contribution of these pathways may vary with gestational age, placental structure and function, maternal and fetal concentration gradients, transporter abundance, genetic variation, and concomitant medications. At present, no transporter genotype, expression threshold, or drug interaction has been validated sufficiently to guide clinical metformin dosing during pregnancy. The principal pharmacological characteristics of metformin relevant to maternal exposure, placental transfer, fetal exposure, and developmental interpretation are summarized in Table 1.

#### Dose, Duration, Gestational Timing, and Maternal–Fetal Exposure

The prescribed maternal dose should not be interpreted as a direct measure of either maternal systemic exposure or the dose received by the fetus. Metformin pharmacokinetics change substantially during pregnancy because of increased glomerular filtration, enhanced tubular secretion, changes in apparent volume of distribution, and gestation-related variation in bioavailability. In a pregnancy pharmacokinetic study, apparent oral clearance, renal clearance, and volume of distribution were increased at both 500 mg and 1000 mg doses. Systemic exposure after a 500mg dose was lower during pregnancy than in nonpregnant women, whereas at the 1000 mg dose, increased estimated bioavailability partly offset the increase in clearance [16]. These nonlinear changes indicate that a proportional increase in the prescribed dose does not necessarily produce a proportional increase in maternal or fetal exposure.

Fetal exposure may nevertheless be substantial. In paired samples collected at delivery, mean umbilical venous and arterial metformin concentrations were approximately 1.4 and 1.5 times the corresponding maternal plasma concentration, respectively, although considerable interindividual variability was present [16]. Concentrations measured at delivery represent only a single time point and cannot reconstruct cumulative exposure throughout gestation. Maternal renal function, adherence, gastrointestinal tolerance, dose titration, dosing interval, time since the last dose, placental function, gestational age, transporter abundance, and the addition of insulin or another glucose-lowering agent may all influence the relationship between the prescribed dose and fetal exposure.

Gestational timing is also biologically relevant because different developmental processes predominate at different stages of pregnancy. Preconception and first-trimester exposure, which is common when metformin is used for PCOS or pregestational T2DM, overlaps with implantation, placentation, and organogenesis. Available human evidence does not indicate a meaningful overall increase in major congenital malformations after first-trimester exposure, but estimates remain imprecise and are affected by heterogeneity of maternal indication and underlying metabolic disease [22]. Reassuring congenital-malformation data should not, however, be interpreted as evidence that early exposure has no effect on placental development, cellular metabolism, or later fetal programming.

Exposure beginning in the second or third trimester overlaps more directly with rapid fetal growth, adipose-tissue development, brain maturation, placental nutrient transport, and increasing fetal metabolic demand. The major randomized trials differed substantially in the timing of treatment initiation. Some PCOS studies continued treatment from preconception or early pregnancy until delivery, trials in T2DM generally initiated or randomized treatment during the first or early second trimester, whereas several GDM trials began metformin only after diagnosis in the late second or third trimester. Consequently, treatment duration may range from a few weeks to nearly the entire pregnancy, and these exposure patterns should not be considered biologically equivalent.

Current trials do not establish a clinically applicable dose–response relationship between metformin and fetal programming. Most studies reported the assigned or maximum tolerated dose but did not repeatedly measure maternal plasma concentrations, fetal concentrations, placental drug accumulation, or cumulative exposure. Dose titration was generally driven by glycemic response and gastrointestinal tolerance, while supplemental insulin was introduced when glucose targets were not achieved. Differences in offspring outcomes among trials therefore cannot be attributed confidently to metformin dose or treatment duration alone because they also reflect maternal indication, disease severity, comparator treatment, glycemic control, placental function, adherence, and the postnatal environment.

From a clinical perspective, metformin should therefore be titrated according to maternal glycemic response, tolerability, renal function, and the need for timely achievement of pregnancy-specific glucose targets, rather than according to an unvalidated assumption that a particular dose is developmentally safer. Failure to achieve glycemic targets should prompt timely initiation or intensification of insulin rather than continued metformin escalation intended solely to avoid injectable treatment. Conversely, the absence of a demonstrated dose-dependent developmental risk should not be interpreted as proof that cumulative fetal exposure is biologically irrelevant. Prospective studies incorporating serial maternal pharmacokinetic sampling, adherence assessment, placental transporter characterization, cord-blood measurement, and long-term offspring follow-up are required to determine whether a clinically meaningful exposure–response relationship exists.

**Figure 2:**
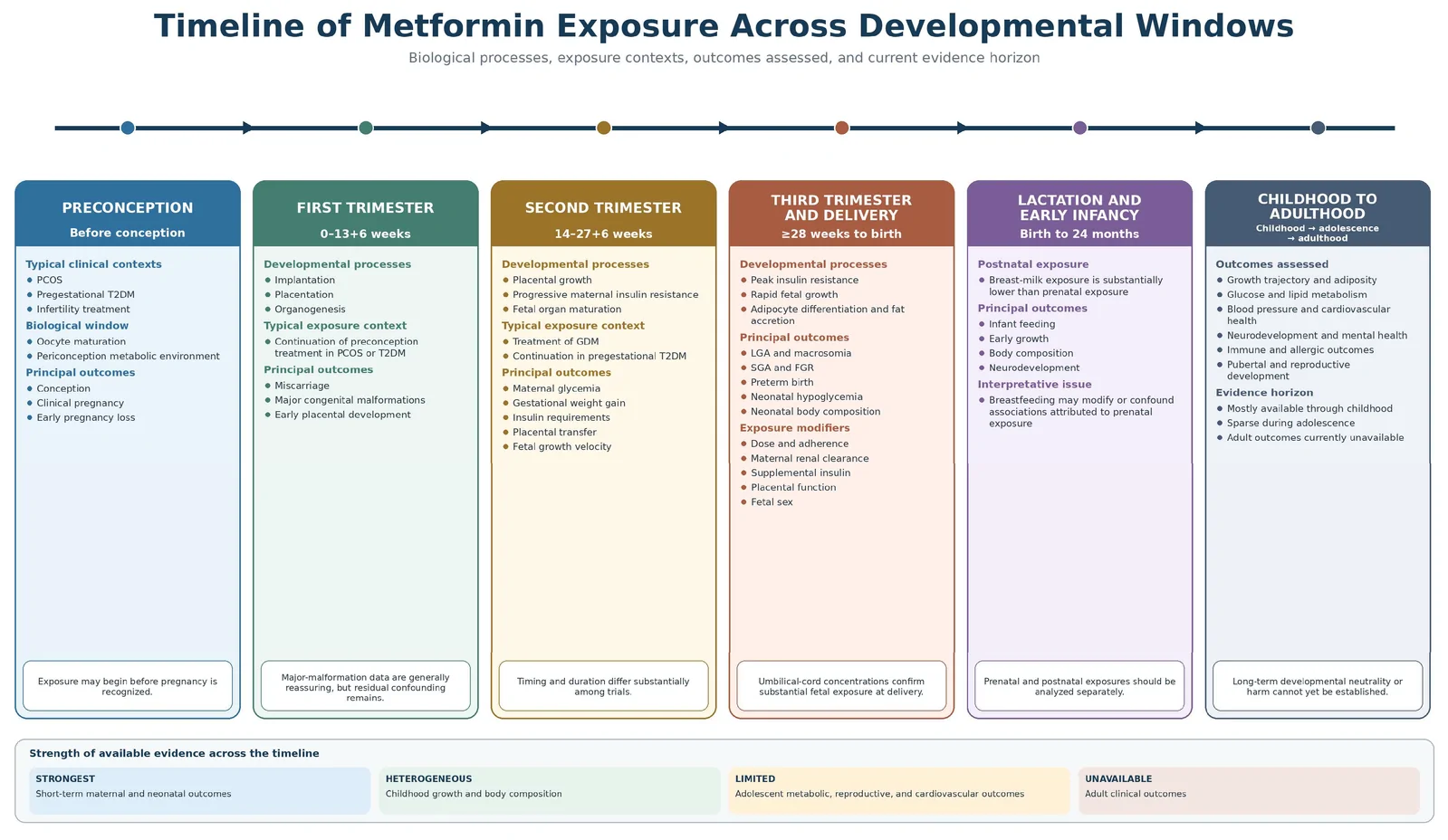
Timeline of metformin exposure across developmental windows and the principal outcomes relevant to developmental interpretation. Exposure before conception and during the first trimester overlaps with oocyte maturation, implantation, placentation, and organogenesis. Second- and third-trimester exposure occurs during progressive maternal insulin resistance, placental maturation, rapid fetal growth, adipocyte differentiation, and metabolic organ development. At delivery, umbilical cord concentrations demonstrate substantial fetal exposure, whereas postnatal exposure through breast milk is considerably lower. Short-term assessment includes maternal glycemic response, gestational weight gain, insulin requirements, congenital malformations, fetal growth, preterm birth, neonatal hypoglycemia, and neonatal body composition. Long-term assessment includes growth trajectory, adiposity, cardiometabolic health, neurodevelopment, immune and allergic outcomes, and reproductive and cardiovascular health. Evidence is strongest for short-term maternal and neonatal outcomes, more limited and heterogeneous for childhood outcomes, sparse during adolescence, and currently insufficient in adulthood.

The developmental windows of metformin exposure, the biological processes occurring during each period, and the principal maternal, fetal, neonatal, and offspring outcomes relevant to interpretation are summarized in Figure 2.

### 3.2. Maternal Metabolic, Placental, and Cellular Pathways

The placenta represents the principal interface through which maternal metabolic disease and pharmacological treatment are translated into fetal signals. In pregnancies complicated by diabetes or obesity, placental function may be altered by hyperglycemia, hyperinsulinemia, dyslipidemia, oxidative stress, inflammation, vascular dysfunction, and excessive nutrient availability. These disturbances may modify placental glucose, amino acid, and lipid transport and contribute to fetal overgrowth, altered body composition, or, in pregnancies with impaired placental reserve, restricted fetal growth [5,6].

Two conceptually distinct pathways should be considered when interpreting the effects of metformin. The first is an indirect maternal pathway, in which improved maternal glycemia, reduced insulin resistance, lower gestational weight gain, and potentially reduced inflammation modify the metabolic environment reaching the placenta and fetus. This pathway is supported by human clinical studies demonstrating maternal metabolic effects of treatment, although the magnitude of benefit varies according to maternal indication and disease severity.

The second is a potential direct placental and fetal pathway resulting from transplacental metformin exposure. Evidence for this pathway is derived predominantly from human placental explants, isolated trophoblasts, animal models, and other experimental systems rather than from clinical studies capable of establishing a causal developmental effect. Consequently, changes in cellular signaling or placental metabolism should be interpreted as evidence of biological plausibility and not as proof of benefit or harm in human offspring.

In primary trophoblasts isolated from term human placentas, exposure to clinically relevant metformin concentrations of 0.01 and 0.1 mmol/L reduced mitochondrial respiration and mitochondrial-dependent ATP production. Markers of oxidative stress were also reduced [23]. These findings indicate that metformin may simultaneously decrease placental energy production and reduce oxidative stress. However, the study was performed in isolated term trophoblasts and cannot determine whether the net effect is beneficial, neutral, or harmful in vivo or during earlier gestational periods.

Other human trophoblast experiments have demonstrated concentration-dependent and fetal sex-specific responses. Reductions in basal, maximal, and ATP-linked respiration were most evident at metformin concentrations of 1 and 10 mmol/L, which exceed the approximately 0.01 mmol/L concentrations generally reported in the maternal circulation during clinical treatment [24]. These findings reinforce the importance of distinguishing effects observed at clinically relevant concentrations from those produced by substantially higher experimental exposures.

AMPK and mTOR constitute central but opposing regulators of cellular energy balance, growth, protein synthesis, and nutrient transport. Metformin-associated AMPK activation may promote adaptive responses to metabolic stress, while subsequent attenuation of mTOR-related signaling may reduce anabolic activity and nutrient transfer. Experimental mouse studies support the ability of metformin to modify placental AMPK–mTOR signaling [25]. Nevertheless, direct evidence that this pathway mediates clinically observed differences in human fetal growth remains limited.

Epigenetic regulation represents another biologically plausible mechanism. In placental tissue from pregnancies complicated by diabetes, fetal sex-specific alterations in histone acetylation and PGC-1α promoter methylation have been reported. In male diabetic placental explants, metformin activated AMPK, increased PGC-1α expression, increased H3K27 acetylation, and reduced PGC-1α promoter methylation [26]. However, these findings originated from a limited explant sample and complementary animal experiments and do not establish a persistent metformin-specific epigenetic signature in the offspring.

More recent in vivo human evidence further emphasizes this uncertainty. In placental samples from 105 women with obesity who had been randomized to metformin or placebo during pregnancy, metformin exposure was not associated with differences in placental gene expression, DNA methylation patterns, telomere length, fibrosis, calcification, or other measures of placental aging [27]. These apparently divergent results may reflect differences in maternal indication, placental metabolic phenotype, fetal sex, exposure timing, metformin concentration, and the biological pathway being examined.

Fetal sex may modify the placental response to maternal metabolic disease and pharmacological exposure. Male and female placentas differ in growth strategy, mitochondrial adaptation, inflammatory signaling, nutrient handling, and responses to metabolic stress. Consequently, an effect observed in the combined study population may conceal different biological responses in pregnancies carrying male and female fetuses. However, fetal sex may also be associated independently with maternal glycemia, fetal size, and pregnancy complications, making it difficult to distinguish a true sex-by-treatment interaction from differences in the underlying maternal–placental phenotype.

Human mechanistic evidence remains limited. Experiments using syncytiotrophoblasts isolated from term placentas demonstrated sexually dimorphic mitochondrial and oxidative responses to metformin. Changes in respiration, glycolytic capacity, proton leak, oxidative stress, and mitochondrial content differed between cells derived from male and female placentas and according to maternal obesity or GDM status [24]. However, several of the most pronounced effects occurred at metformin concentrations of 1–10 mmol/L, substantially exceeding the approximately 0.01 mmol/L concentrations generally reported in the maternal circulation during clinical treatment. These findings therefore demonstrate biological responsiveness rather than a clinically established sex-specific effect.

Sex-dependent epigenetic findings have also been reported in placentas from pregnancies complicated by diabetes. In a small study of human placental tissue and explants, maternal diabetes was associated with altered H3K27 acetylation and PGC-1α promoter methylation, particularly in male placentas. Metformin exposure of male diabetic placental explants activated AMPK, increased PGC-1α expression and H3K27 acetylation, and reduced PGC-1α promoter methylation [26]. Because these observations were derived from a limited number of term placental samples and were supported partly by complementary mouse experiments, they do not establish a persistent sex-specific epigenetic effect in human offspring.

Human clinical evidence is only beginning to emerge. A secondary analysis of the randomized EMERGE trial evaluated whether fetal sex modified maternal glycemic and neonatal responses to early metformin treatment for GDM [28]. Among pregnancies carrying male fetuses, metformin exposure was associated with lower fasting glucose at 32 and 38 weeks and less frequent insulin use than placebo, while the corresponding differences were smaller in pregnancies carrying female fetuses. Conversely, birthweight below 2500 g occurred more frequently among metformin-exposed female than placebo-exposed female neonates, whereas this pattern was not observed among males. Nevertheless, the formal sex-by-treatment interactions were not statistically significant. These subgroup findings should therefore be considered hypothesis-generating and should not currently guide sex-specific treatment selection.

Animal studies provide further evidence that fetal sex may influence developmental responses, but the direction of effect has not been consistent. In a mouse model of obese glucose-intolerant pregnancy, maternal metformin treatment was associated with different patterns of adiposity, adipose tissue inflammation, hepatic lipid accumulation, hyperleptinemia, and hyperinsulinemia in male and female offspring, with effects also varying according to offspring age [29]. Such findings indicate that fetal sex and later-life environmental exposure may jointly modify the observed phenotype, but they cannot be extrapolated directly to human pregnancy.

Overall, the evidence that fetal sex modifies clinically relevant responses to prenatal metformin exposure remains of low or exploratory confidence. Most pregnancy trials were not designed or powered for sex-by-treatment interaction testing, sex-stratified offspring samples are small, and positive subgroup findings are vulnerable to multiple testing and attrition. Future trials and follow-up studies should prespecify fetal sex as a potential effect modifier, report interaction tests rather than separate within-sex comparisons alone and retain sufficient numbers of male and female offspring for long-term metabolic, cardiovascular, neurodevelopmental, and reproductive assessment.

Animal studies provide additional evidence of biological plausibility but have not identified a uniform developmental phenotype. A systematic review and meta-analysis of 37 animal studies, including 3133 offspring from 716 L, found no significant overall effect of prenatal metformin exposure on anthropometric, cardiovascular, or metabolic offspring outcomes. Approximately 95% of the included studies used rodent models; between-study heterogeneity was high, and the risk of bias was frequently unclear because of incomplete methodological reporting [30]. Representative mouse studies have reported both adverse and apparently beneficial long-term metabolic phenotypes, depending on the maternal metabolic model and the postnatal high-fat-diet challenge [31,32]. In one model involving metabolically healthy dams, prenatal metformin exposure was followed by greater weight gain, increased mesenteric fat, and impaired glucose tolerance in male offspring challenged with a high-fat diet during adulthood. Conversely, in a maternal high-fat-diet model, prenatal metformin exposure was associated with lower offspring weight and adiposity and preservation of glucose tolerance during a later high-fat-diet challenge. These apparently divergent findings indicate that experimental outcomes depend on maternal metabolic status, offspring sex, exposure conditions, and the postnatal nutritional environment. Animal findings should therefore be regarded as mechanistic and hypothesis-generating and should not be extrapolated directly to human pregnancy.

Overall, evidence that metformin improves the maternal metabolic environment is of higher confidence than evidence for direct placental or fetal programming effects. Human placental findings provide moderate-to-low confidence regarding short-term cellular responses, whereas proposed AMPK–mTOR, mitochondrial, and epigenetic pathways remain predominantly mechanistic and exploratory. Current evidence does not demonstrate that metformin produces a persistent and clinically relevant molecular signature in human offspring. The principal maternal–placental–fetal pathways potentially associated with metformin exposure and the certainty of the supporting evidence are summarized in Table 2.

Narrative confidence descriptors were assigned according to the framework defined in Section 2.6 and do not represent a formal GRADE assessment. Mechanistic and experimental findings indicate biological plausibility but should not be interpreted as proof of causal clinical effects in human offspring.

**Figure 3:**
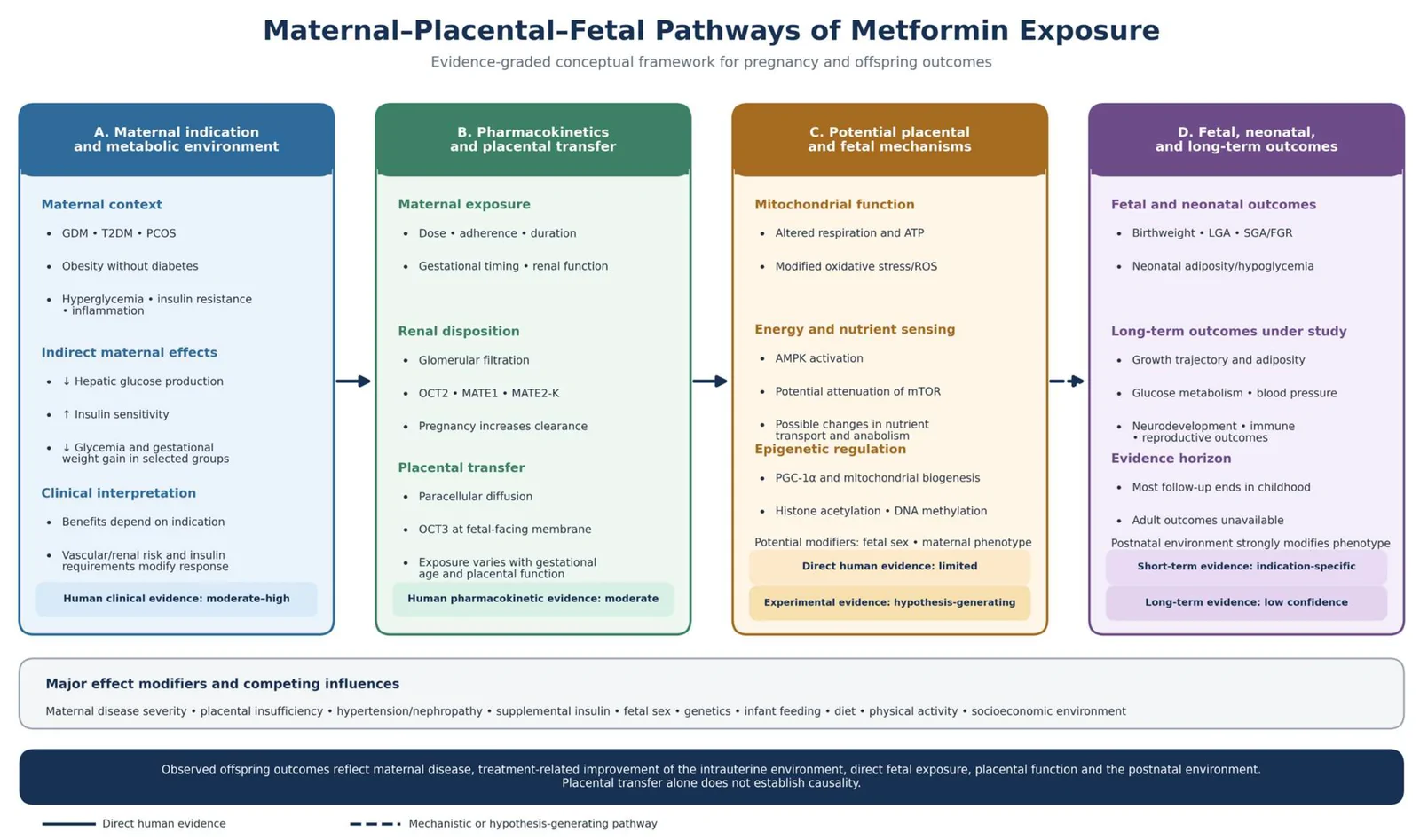
Proposed maternal–placental–fetal pathways linking metformin exposure with pregnancy and offspring outcomes. (A) Maternal indication and metabolic environment, including the underlying metabolic disorder, indirect maternal effects of metformin, and indication-dependent clinical considerations. (B) Maternal pharmacokinetics and placental transfer, including the influence of dose, adherence, gestational timing, renal function, renal transporters, paracellular diffusion, and OCT3-mediated fetal disposition. (C) Potential placental and fetal mechanisms involving mitochondrial function, oxidative stress, AMPK–mTOR signaling, nutrient sensing, mitochondrial biogenesis, and epigenetic regulation. (D) Fetal, neonatal, and long-term offspring outcomes, together with the current limitations of the available evidence.Metformin may influence fetal development indirectly by improving maternal glycemia, insulin resistance, gestational weight gain, and the maternal metabolic environment, and directly following placental transfer and fetal exposure. Maternal metformin concentrations are influenced by dose, treatment duration, adherence, gestational timing, renal function, and renal transport through OCT2, MATE1, and MATE2-K. Placental transfer appears to involve paracellular diffusion and OCT3-mediated transport, although the relative contribution of individual transporters in human pregnancy remains incompletely defined. Proposed downstream mechanisms include modification of mitochondrial respiration, oxidative stress, AMPK–mTOR signaling, nutrient sensing, mitochondrial biogenesis, and epigenetic regulation. These pathways are biologically plausible but are supported predominantly by human placental experiments, in vitro studies, and animal models rather than validated causal evidence in human offspring. Solid arrows indicate relationships supported by direct human clinical or pharmacokinetic evidence, whereas dashed arrows indicate mechanistic or hypothesis-generating relationships. Maternal indication, disease severity, vascular and renal comorbidity, placental function, fetal sex, supplemental insulin, and the postnatal environment may modify the observed outcomes.

The proposed maternal, pharmacokinetic, placental, and fetal pathways through which metformin exposure may influence pregnancy and offspring outcomes are summarized in Figure 3. The figure distinguishes pathways supported by direct human clinical or pharmacokinetic evidence from mechanistic associations derived predominantly from human placental experiments, in vitro studies, and animal models. Importantly, placental transfer and biological plausibility do not establish either developmental toxicity or developmental neutrality.

Figure 3 should therefore be interpreted as an evidence-graded conceptual model rather than as a proven causal sequence. Human evidence directly supports maternal metabolic effects, altered pregnancy pharmacokinetics, placental transfer, and substantial fetal exposure. By contrast, the links between fetal metformin concentrations, placental cellular signaling, and persistent offspring phenotypes remain insufficiently established. The absence of detectable differences in placental gene expression, DNA methylation, telomere length, fibrosis, or calcification in a randomized human placental study further demonstrates that individual experimental mechanisms cannot be assumed to operate uniformly in vivo [27].

### 3.3. Clinical Evidence and Offspring Outcomes

Available human evidence has not demonstrated a clear increase in major congenital malformations after first-trimester metformin exposure. However, interpretation remains complicated by the underlying maternal indication, particularly pregestational diabetes, which independently increases congenital-anomaly risk.

#### 3.3.1. Gestational Diabetes Mellitus

GDM is the maternal indication for which metformin has been most extensively evaluated. Contemporary clinical guidance continues to regard insulin as the preferred pharmacological treatment when lifestyle intervention is insufficient, particularly because insulin does not cross the placenta and can be adjusted rapidly to the maternal glucose profile [13]. Metformin is nevertheless used in selected women because it is orally administered, inexpensive, generally acceptable to patients, and commonly associated with less maternal gestational weight gain. Its use should not delay insulin initiation when glycemic targets are not achieved.

The MiG trial randomized 751 women with GDM to metformin, with supplemental insulin when required, or to insulin alone [33]. The primary composite neonatal outcome occurred in 32.0% of the metformin group and 32.2% of the insulin group (RR 0.99, 95% CI 0.80–1.23). However, 46.3% of women allocated to metformin required supplemental insulin. This finding demonstrates that metformin-based treatment was not equivalent to metformin monotherapy and that a substantial proportion of women could not achieve adequate glycemic control without insulin. Treatment acceptability was higher with metformin: 76.6% of women allocated to metformin reported that they would choose the same treatment again, compared with 27.2% allocated to insulin.

More recent randomized evidence reinforces the need to distinguish metformin from broader sequential oral-treatment strategies. In the SUGAR-DIP trial, 79% of participants assigned to oral treatment maintained glycemic control without insulin, but the strategy of metformin followed by glyburide when required did not meet the prespecified criterion for noninferiority to insulin for prevention of large-for-gestational-age birth [34]. Large-for-gestational-age infants occurred in 23.9% of the oral-treatment group and 19.9% of the insulin group, corresponding to an absolute risk difference of 4.0% (95% CI −1.7% to 9.8%). Because glyburide formed part of the sequential intervention, these findings should not be attributed to metformin alone.

Meta-analytic evidence generally supports comparable glycemic control and selected short-term maternal and neonatal advantages with metformin-based treatment. A recent meta-analysis of ten randomized trials reported a lower risk of neonatal hypoglycemia with metformin than with insulin (OR 0.65, 95% CI 0.46–0.92) and a modest reduction in mean birthweight (mean difference −68.96 g, 95% CI −108.34 to −29.57) [35]. Earlier analyses similarly reported lower neonatal birthweight, lower maternal gestational weight gain, and reduced risks of macrosomia or large-for-gestational-age birth. These short-term findings are supported by randomized evidence but remain affected by differences in GDM diagnostic criteria, treatment dose, timing of initiation, supplemental insulin use, and outcome definitions.

Lower mean birthweight should not automatically be interpreted as either beneficial or harmful. A 2019 meta-analysis found that neonates exposed to metformin were, on average, 107.7 g lighter than those exposed to insulin (95% CI −182.3 to −32.7), with lower odds of macrosomia (OR 0.59, 95% CI 0.46–0.77) and large-for-gestational-age birth (OR 0.78, 95% CI 0.62–0.99), but no significant difference in small-for-gestational-age birth [36]. The high heterogeneity for birthweight, including an I^2^ value of 83%, indicates that the magnitude of the growth effect varied substantially among studies.

Long-term offspring findings are less certain. At two years of age, the MiG TOFU study identified larger mid-upper arm circumference and selected skinfold measurements in metformin-exposed children, but no difference in total fat mass or body-fat percentage [37]. At seven years, the Adelaide cohort showed no clear differences in anthropometric or metabolic outcomes. At nine years, children in the Auckland metformin group were larger according to several anthropometric measures, including weight and waist circumference, but total and abdominal body-fat percentages and measured metabolic biomarkers were similar between treatment groups [38]. Follow-up was incomplete and differed considerably between centers, with 109 of 181 eligible children assessed in Adelaide and 99 of 396 in Auckland. These differences increase the possibility of attrition and selection bias.

The 2019 growth meta-analysis reported that metformin-exposed infants were approximately 440 g heavier at 18–24 months (95% CI 50–830 g) and that BMI in mid-childhood was 0.78 kg/m^2^ higher than after insulin exposure (95% CI 0.23–1.33) [36]. However, the childhood analysis included only 520 children from three studies, and the clinical meaning of this modest BMI difference remains unclear. A single BMI measurement cannot establish an adverse developmental trajectory, and greater childhood size may represent transient catch-up growth, population-specific growth, or residual effects of the maternal and postnatal environment rather than a direct metformin effect.

Complementary observational evidence is broadly reassuring but does not eliminate uncertainty. In a Finnish register-based cohort, metformin exposure was not associated with increased risks of childhood obesity, disturbances of glucose regulation, diabetes, hypertension, PCOS, or motor-social developmental problems compared with insulin [39]. However, metformin exposure was associated with higher odds of small-for-gestational-age birth (IPTW-adjusted OR 1.65, 95% CI 1.16–2.34). The cohort included different maternal indications and treatment combinations, and residual confounding by disease severity and indication remains possible.

Overall, the confidence of evidence is high-to-moderate for comparable short-term glycemic efficacy and a lower risk of neonatal hypoglycemia, and moderate for modestly lower birthweight and reduced fetal overgrowth. Confidence is low for childhood growth and cardiometabolic consequences because follow-up samples are small, attrition is substantial, findings differ across geographic cohorts, and most children have not been followed through puberty or adulthood. Current evidence therefore does not establish either long-term developmental harm or developmental neutrality after metformin treatment for GDM.

#### 3.3.2. Pregestational Type 2 Diabetes Mellitus

Pregnancies complicated by pregestational T2DM have a high baseline risk of congenital anomalies, hypertensive disorders, fetal overgrowth, stillbirth, preterm birth, neonatal hypoglycemia, and later offspring metabolic disease. Insulin therefore remains the principal pharmacological treatment, while metformin has been evaluated mainly as an adjunct rather than as a complete substitute. Interpretation is particularly complex because maternal obesity, chronic hyperglycemia, insulin resistance, vascular disease, nephropathy, and placental dysfunction may independently influence fetal growth and neonatal outcomes.

The MiTy trial randomized 502 women with T2DM receiving insulin to additional metformin at a dose of 1000 mg twice daily or placebo between 6 and 22 + 6 weeks of gestation [40]. The primary composite fetal and neonatal outcome occurred in 40% of both groups (RR 1.02, 95% CI 0.83–1.26), indicating that the addition of metformin did not reduce overall neonatal morbidity.

Several prespecified maternal outcomes nevertheless favored metformin. At 34 weeks, mean HbA1c was 5.90% in the metformin group and 6.10% in the placebo group, while mean glucose was lower by approximately 0.2 mmol/L. Women receiving metformin required less insulin, with mean daily doses of 1.1 versus 1.5 units/kg, corresponding to a difference of −0.4 units/kg/day (95% CI −0.5 to −0.2). Gestational weight gain was also lower by 1.8 kg (95% CI −2.7 to −0.9), and cesarean birth occurred less frequently (53% versus 63%; RR 0.85, 95% CI 0.73–0.99). Hypertensive disorders occurred in 23% of both groups and were not reduced by metformin.

Neonates exposed to metformin were, on average, 218 g lighter at birth than those exposed to placebo in addition to insulin (mean difference −218 g, 95% CI −353 to −82). Fewer neonates had a birthweight above the 97th percentile (9% versus 15%; RR 0.58, 95% CI 0.34–0.97) or weighed at least 4000 g (12% versus 19%; RR 0.65, 95% CI 0.43–0.99). Neonatal skinfold thickness and fat mass were also lower. These findings support a reduction in excessive fetal growth and neonatal adiposity. However, SGA birth occurred more frequently with metformin than with placebo (13% versus 7%; RR 1.96, 95% CI 1.10–3.64) [40].

SGA and fetal growth restriction should not be treated as interchangeable terms. SGA is an anthropometric classification, usually defined by birthweight below the 10th percentile, and includes both constitutionally small infants and infants affected by pathological growth restriction. FGR refers to failure of the fetus to achieve its biological growth potential, commonly because of placental dysfunction, and is evaluated using longitudinal growth, fetal biometry, growth velocity, Doppler findings, and the wider maternal and placental context [41]. Because the MiTy outcome was based primarily on birthweight centile, the trial could not determine whether every SGA infant had placental FGR or was constitutionally small.

A secondary MiTy analysis identified both metformin exposure and maternal vascular or renal comorbidity as predictors of SGA [42]. Metformin use was associated with an adjusted OR of 2.26 (95% CI 1.19–4.74), while chronic hypertension and/or nephropathy was associated with an OR of 3.05 (95% CI 1.58–5.81). Among women receiving metformin, the absolute frequency of SGA was 25.0% in those with chronic hypertension or nephropathy, compared with 9.8% in those without these comorbidities. SGA infants in the metformin group were delivered later than SGA infants in the placebo group, at a mean of 37.2 versus 35.3 weeks. This finding does not establish that metformin-associated SGA represented a more severe neonatal phenotype, but it reinforces the importance of maternal vascular disease and placental reserve when interpreting the growth effect.

Complementary evidence was provided by the MOMPOD trial, which included women with preexisting T2DM or diabetes diagnosed before 23 weeks of gestation. Adding metformin 1000 mg twice daily to insulin did not significantly reduce the composite adverse neonatal outcome, which occurred in 71% of the metformin group and 74% of the placebo group (adjusted OR 0.86, 95% CI 0.63–1.19) [43]. Metformin was associated with lower odds of large-for-gestational-age birth (adjusted OR 0.63, 95% CI 0.46–0.86). Because the trial combined preexisting T2DM and diabetes diagnosed early in pregnancy, its results cannot be attributed exclusively to pregestational T2DM. Nevertheless, it supports the conclusion that reduced fetal overgrowth does not necessarily translate into improvement in a broad composite neonatal outcome.

The MiTy Kids follow-up included 283 of 465 eligible children, corresponding to a follow-up rate of approximately 61% [44]. At 24 months, mean BMI Z score was similar after metformin and placebo exposure (0.84 versus 0.91; mean difference 0.07, 95% CI −0.31 to 0.45), as was the sum of skinfold thicknesses (23.0 versus 23.8 mm; mean difference 0.8 mm, 95% CI −0.7 to 2.3). No overall difference in BMI trajectory was identified. A treatment-related difference in BMI trajectory among boys, with higher BMI between 6 and 24 months after metformin exposure, was exploratory and requires confirmation. Follow-up to two years cannot address outcomes emerging during later childhood, puberty, reproductive life, or adulthood.

The clinical interpretation should therefore be phenotype-specific. In women with marked insulin resistance, excessive gestational weight gain, high insulin requirements, and fetal overgrowth, adjunctive metformin may provide maternal metabolic and fetal-size benefits. In women with chronic hypertension, nephropathy, impaired renal function, suspected placental insufficiency, declining fetal growth velocity, or established FGR, the balance may be less favorable and routine addition or continuation of metformin requires particular caution.

Monitoring should include maternal renal function, glycemic response, insulin requirements, blood pressure, and serial third-trimester assessment of fetal biometry and growth velocity. When estimated fetal weight or abdominal circumference falls below the 10th percentile, growth velocity decreases, or placental dysfunction is suspected, umbilical artery Doppler and additional fetal surveillance should be performed according to established FGR guidance. The indication for continued metformin treatment should be reassessed when emerging FGR, abnormal Doppler findings, pre-eclampsia, or other evidence of impaired placental function is identified.

Overall, confidence is high-to-moderate that adjunctive metformin reduces maternal insulin requirements, gestational weight gain, and excessive fetal growth in T2DM. Confidence is moderate regarding an increased SGA signal because it was observed in a large randomized trial and was stronger in women with chronic hypertension or nephropathy. Confidence regarding long-term offspring outcomes remains low because follow-up is limited to 24 months, attrition was substantial, and outcomes after puberty and in adulthood are unavailable.

#### 3.3.3. Polycystic Ovary Syndrome

Metformin exposure in women with PCOS may begin before conception as part of metabolic or fertility treatment and may subsequently be discontinued in early pregnancy or continued until delivery. These exposure patterns should be distinguished because preconception treatment intended to improve ovulation or pregnancy achievement addresses a different clinical question from routine continuation throughout pregnancy. PCOS is itself heterogeneous and may involve obesity, insulin resistance, hyperandrogenism, chronic low-grade inflammation, anovulatory infertility, and the use of ovulation-induction or assisted reproductive treatments. These factors independently influence miscarriage, preterm birth, GDM, fetal growth, and long-term offspring metabolic risk.

The PregMet2 trial randomized 487 women with PCOS to metformin or placebo from the late first trimester until delivery [45]. Metformin was administered at 500 mg twice daily during the first treatment week and subsequently increased to 1000 mg twice daily. The composite primary outcome of late miscarriage or preterm birth occurred in 5% of women receiving metformin and 10% receiving placebo (OR 0.50, 95% CI 0.22–1.08; *p* = 0.08). Although the point estimate suggested a possible benefit, the confidence interval included no effect and the primary outcome was not statistically significant.

In a post hoc individual-participant-data analysis combining PregMet2 with two previous PCOS trials, late miscarriage or preterm birth occurred in 5% of metformin-treated pregnancies and 10% of placebo-treated pregnancies (OR 0.43, 95% CI 0.23–0.79) [45]. This pooled finding supports a possible reduction in late pregnancy loss or preterm delivery, but it should be interpreted as secondary evidence because the analysis was post hoc and combined trials conducted during different periods.

Metformin did not prevent GDM in PregMet2. GDM occurred in 25% of the metformin group and 24% of the placebo group (OR 1.09, 95% CI 0.69–1.66). No clear reduction in pre-eclampsia, pregnancy-induced hypertension, or neonatal intensive care admission was demonstrated. Consequently, routine continuation of metformin throughout pregnancy solely to prevent GDM is not supported by randomized evidence. Similarly, the evidence does not justify universal continuation solely to prevent miscarriage in all women with PCOS, particularly when the individual patient has no clear metabolic or obstetric indication.

Offspring follow-up has generated a more specific growth signal. In a follow-up of two randomized PCOS trials including 182 children, offspring exposed to metformin at doses of 1700–2000 mg/day had a higher mean weight Z score at four years than placebo-exposed children, with a between-group difference of 0.38 (95% CI 0.07–0.69) [46]. BMI Z score was also higher by 0.45 (95% CI 0.11–0.78). Overweight or obesity was identified in 32% of metformin-exposed children and 18% of placebo-exposed children (OR 2.17, 95% CI 1.04–4.61). Height did not differ significantly between groups.

The PedMet follow-up assessed 141 of 255 eligible children from the original PregMet trial at 5–10 years, corresponding to participation of approximately 55% [47]. Mean BMI Z score remained higher after prenatal metformin exposure, with a between-group difference of 0.41 (95% CI 0.03–0.78). This finding suggests that the earlier difference in body size may persist into later childhood, but substantial attrition limits certainty and raises the possibility of selection bias. A higher BMI Z score alone does not establish adverse metabolic programming, particularly without consistent evidence of impaired glucose metabolism, dyslipidemia, hypertension, or abnormal body-fat distribution.

Causal interpretation remains difficult even in randomized follow-up studies. Randomization reduces baseline confounding, but long-term childhood outcomes may still be influenced by differential follow-up, maternal BMI, maternal glycemia and androgen profile, gestational weight gain, breastfeeding, parental adiposity, childhood diet, physical activity, socioeconomic conditions, and shared genetic susceptibility. PCOS-related hyperandrogenism and maternal metabolic dysfunction may themselves affect offspring growth and endocrine development independently of metformin exposure.

Observational studies are additionally vulnerable to confounding by indication. Women who continue metformin during pregnancy may differ systematically from those who discontinue it in the severity of insulin resistance, obesity, infertility history, previous miscarriage, access to fertility treatment, and clinician-perceived obstetric risk. Associations identified in such studies should therefore not be attributed automatically to a direct drug effect unless these differences are addressed adequately.

The clinical decision should distinguish three scenarios: metformin used before conception to support metabolic management or fertility treatment; short continuation during early pregnancy while the treatment plan is reassessed; and prolonged exposure until delivery. The indication, dose, timing, maternal metabolic phenotype, fertility treatment, and duration of exposure should be documented explicitly. Continued treatment may be considered when an individualized maternal metabolic or obstetric benefit is expected, but it should not be automatic solely because conception occurred while the woman was taking metformin.

Overall, confidence is moderate that metformin does not prevent GDM in women with PCOS. Confidence is low-to-moderate regarding a reduction in late miscarriage or preterm birth because the primary PregMet2 result was imprecise and the statistically significant finding arose from a post hoc pooled analysis. Confidence is low-to-moderate regarding higher childhood BMI because the signal was observed in randomized follow-up cohorts but was affected by limited sample size, approximately 45% attrition in PedMet, and insufficient follow-up into adolescence and adulthood.

#### 3.3.4. Maternal Obesity Without Diabetes

Metformin has been investigated in pregnant women with overweight or obesity but without diabetes on the hypothesis that reducing insulin resistance and maternal gestational weight gain might improve placental function, reduce excessive fetal growth, and modify the offspring obesity trajectory. This population differs fundamentally from women with GDM or T2DM because there is no established hyperglycemic indication for pharmacological treatment. Consequently, any maternal benefit must be weighed against direct fetal drug exposure in the absence of proven improvement in clinically important pregnancy or neonatal outcomes.

The EMPOWaR trial randomized 449 women with a BMI of at least 30 kg/m^2^ and normal glucose tolerance to metformin, titrated to a maximum of 2500 mg/day, or placebo from 12 to 16 weeks of gestation until delivery [48]. Mean birthweight was almost identical between groups: 3462 g after metformin and 3463 g after placebo. Metformin did not alter the gestational-age-, parity-, and fetal-sex-standardized birthweight Z score (adjusted mean difference −0.029, 95% CI −0.217 to 0.158). The trial therefore provided no evidence that metformin reduced fetal size or improved the principal pregnancy outcome in obese women without diabetes.

A separate randomized trial included women without diabetes who had a BMI greater than 35 kg/m^2^ and compared metformin at 3 g/day with placebo from 12 to 18 weeks until delivery [49]. Median neonatal birthweight Z score did not differ significantly between the metformin and placebo groups (0.05 versus 0.17; *p* = 0.66). Metformin reduced median gestational weight gain from 6.3 to 4.6 kg. The incidence of pre-eclampsia was also lower in this individual trial (3.0% versus 11.3%; OR 0.24, 95% CI 0.10–0.61), but gastrointestinal and other treatment-related adverse effects were more frequent. No significant differences were observed in GDM, large-for-gestational-age birth, or the principal neonatal outcomes.

The apparently favorable pre-eclampsia finding should be interpreted cautiously because it was a secondary outcome and was not reproduced consistently across the broader trial evidence. Differences between the obesity trials in baseline BMI, ethnicity, treatment dose, adherence, co-interventions, diagnostic testing for GDM, and outcome definitions may explain part of the inconsistency. A statistically significant secondary outcome from a single trial should therefore not be treated as proof that metformin prevents pre-eclampsia in obese women without diabetes.

The GRoW trial evaluated metformin, at a maximum dose of 2000 mg/day, in addition to structured dietary and lifestyle advice among 524 women with a BMI of at least 25 kg/m^2^ [50]. Birthweight above 4000 g occurred in 16% of the metformin group and 14% of the placebo group (adjusted RR 0.97, 95% CI 0.65–1.47). Metformin reduced average weekly gestational weight gain by 0.08 kg (95% CI −0.14 to −0.02), but did not improve total gestational weight gain, GDM incidence, major pregnancy outcomes, neonatal outcomes, maternal quality of life, or emotional well-being. Women receiving metformin were more likely to gain less weight than recommended (adjusted RR 1.46, 95% CI 1.10–1.94), indicating that reduced weight gain should not automatically be interpreted as a clinical benefit.

Follow-up of children from the GRoW trial did not demonstrate a long-term childhood benefit [51]. The proportion of children with a BMI Z score above the 85th percentile was similar after prenatal metformin and placebo exposure at six months, 18 months, and three to five years. More than half of the assessed children in both groups had a BMI Z score above the 85th percentile at 18 months and at three to five years, suggesting that maternal obesity and the shared postnatal environment remained dominant determinants of childhood obesity risk. Prenatal metformin treatment did not appear to overcome this underlying risk.

These trials also illustrate the limitations of using gestational weight gain as a surrogate for meaningful maternal or offspring benefit. A modest reduction in maternal weight gain does not necessarily lead to lower neonatal adiposity, reduced obstetric morbidity, or improved childhood body composition. Conversely, excessively restricted gestational weight gain may be undesirable in some women and should not be pursued without attention to nutritional adequacy, maternal metabolic health, and fetal growth.

Obesity is biologically heterogeneous. Some women have marked insulin resistance, dysglycemia, metabolic dysfunction, or subsequently develop GDM, whereas others remain normoglycemic. Once GDM or overt diabetes is diagnosed, metformin should be evaluated according to the evidence and recommendations for that specific metabolic indication rather than continued as treatment of obesity itself. Obesity alone should not be treated as equivalent to diabetes or used as an indication for routine fetal metformin exposure.

Lifestyle and nutritional care, appropriate gestational weight-gain counseling, screening for hyperglycemia and hypertensive disorders, fetal growth assessment according to obstetric risk, and preconception or postpartum weight-management strategies remain the principal clinical approaches. Metformin should not be initiated routinely during pregnancy solely to reduce maternal weight gain, prevent fetal overgrowth, or prevent future obesity in the child.

Overall, confidence is high that metformin does not meaningfully reduce neonatal birthweight or major neonatal growth outcomes in women with obesity but without diabetes. Confidence is moderate that it may modestly reduce maternal gestational weight gain, although the clinical importance of this effect is uncertain. Confidence is low regarding prevention of pre-eclampsia and long-term offspring benefit because findings are inconsistent, secondary outcomes are imprecise, and available childhood follow-up has not demonstrated a clear advantage. The principal randomized trials across the maternal indications discussed above and their relevance to maternal, fetal, and developmental interpretation are summarized in Table 3.

Assigned dose and maximum permitted dose should not be interpreted as equivalent to maternal or fetal exposure because pregnancy alters metformin clearance, bioavailability, distribution, and placental transfer, while adherence and treatment duration vary substantially among trials.

Trials are grouped by maternal indication rather than ranked according to treatment efficacy. Differences in comparator, gestational timing, glycemic targets, supplemental insulin, cointerventions, primary outcome definition, and baseline maternal metabolic and vascular risk preclude direct numerical comparison across trials.

Taken together, the randomized evidence does not support a single indication-independent interpretation of metformin exposure. In GDM, metformin-based treatment may provide acceptable glycemic control and reduce treatment burden in selected women, although supplemental insulin or additional medication is frequently required. In T2DM, maternal metabolic benefits and reduced excessive fetal growth must be weighed against the SGA signal observed in MiTy and the absence of improvement in broad neonatal composite outcomes in MiTy and MOMPOD. In PCOS, a possible reduction in late miscarriage or preterm birth remains uncertain, while prevention of GDM has not been demonstrated. In pregnancies complicated by maternal overweight or obesity without diabetes, the principal randomized trials have not demonstrated consistent fetal or neonatal benefit.

#### 3.3.5. Confounding by Indication and Challenges in Causal Interpretation

Causal interpretation of offspring outcomes after prenatal metformin exposure is complicated by confounding by indication. Metformin is not prescribed randomly across the pregnant population but is used in women with GDM, pregestational T2DM, PCOS, obesity, or combinations of these conditions. Each indication is independently associated with a distinct maternal metabolic, hormonal, vascular, inflammatory, and placental environment that may influence fetal growth and long-term offspring health. Therefore, comparisons that classify pregnancies only as metformin-exposed or unexposed may be attributed to the drug effects that arise partly or predominantly from the underlying maternal condition.

The direction of confounding may differ according to the comparator. Women treated with insulin frequently have earlier diagnosis, greater hyperglycemia, higher BMI, longer diabetes duration, higher insulin requirements, or more severe metabolic disease than women managed with metformin alone. Conversely, women whose GDM remains controlled by lifestyle intervention generally have milder hyperglycemia than those requiring pharmacological treatment. Metformin-versus-insulin and metformin-versus-diet comparisons therefore estimate different clinical contrasts and cannot be interpreted as equivalent assessments of fetal drug exposure.

This problem is illustrated by Finnish register-based studies. Metformin exposure was associated with an increased risk of SGA when compared with insulin exposure, with an inverse-probability-weighted OR of 1.65 (95% CI 1.16–2.34) [39]. However, a subsequent analysis comparing metformin-exposed pregnancies with pregnancies involving non-pharmacologically treated GDM found no increased SGA risk, with a weighted OR of 1.01 (95% CI 0.75–1.37) in the GDM-restricted analysis [53]. The difference according to comparator suggests that treatment selection, disease severity, residual hyperglycemia, and other unmeasured maternal characteristics contribute materially to the observed association. It does not exclude a metformin-related effect on fetal growth, but it prevents the SGA finding from being interpreted as a uniform direct pharmacological effect across all indications.

Placental dysfunction represents an additional source of both effect modification and confounding. Chronic hypertension, diabetic nephropathy, vascular disease, pre-eclampsia, impaired placental perfusion, and emerging fetal growth restriction may independently reduce fetal growth. These conditions may also influence the decision to initiate, continue, intensify, or discontinue metformin. In MiTy, the higher overall SGA rate among metformin-exposed infants was particularly relevant because the maternal population had pregestational T2DM and included women with substantially greater vascular and placental risk than most GDM cohorts. A reduction in fetal size in this setting should therefore be interpreted differently from a modest reduction in excessive fetal growth among women with uncomplicated late-diagnosed GDM.

Supplemental insulin creates further interpretative complexity. Women requiring insulin in addition to metformin generally have poorer glycemic control or more severe insulin resistance than women controlled with metformin alone. Outcomes in the combination-treatment group consequently reflect both exposure to two treatments and the metabolic severity that prompted treatment escalation. In the Finnish register study, combination treatment was associated with higher risks of LGA, preterm birth, and neonatal hypoglycemia; the investigators considered confounding by indication or disease severity a plausible explanation [39]. Such findings should not be interpreted as evidence that metformin and insulin interact pharmacologically to produce these outcomes unless treatment severity and time-varying glycemic control are addressed adequately.

Randomization reduces baseline confounding in the original pregnancy trial but does not fully identify the direct developmental effect of metformin. The randomized treatment assignment produces a total treatment effect that includes direct fetal drug exposure and indirect effects mediated through improved maternal glycemia, altered gestational weight gain, reduced insulin dose, differences in fetal growth, and changes in obstetric management. Long-term follow-up may additionally lose the protection of randomization when participation is incomplete or differs according to maternal characteristics, treatment group, geographic site, or offspring health. Consequently, an association observed during childhood cannot automatically be assigned to a direct fetal cellular effect of metformin.

Maternal obesity and inherited susceptibility remain particularly important for childhood adiposity. A recent meta-analysis found no overall difference in BMI at the oldest available childhood follow-up between metformin-exposed and unexposed children (standardized mean difference −0.02, 95% CI −0.11 to 0.07), but the certainty of evidence was low and results varied according to offspring age and maternal indication [54]. A higher BMI was observed in the PCOS subgroup, whereas no corresponding difference was identified in the diabetes or obesity subgroups. This variation supports indication-specific interpretation and cautions against pooling biologically different maternal populations into a single estimate of developmental risk.

The postnatal environment may further amplify, attenuate, or obscure intrauterine effects. Breastfeeding, infant feeding practices, childhood diet, physical activity, socioeconomic circumstances, parental BMI, and shared genetic susceptibility all influence growth and cardiometabolic outcomes. These factors are incompletely measured in many follow-up studies and may differ among families who participate in long-term assessment. Accordingly, the observed childhood phenotype should be interpreted as the product of maternal disease, treatment-related improvement of the intrauterine environment, direct fetal exposure, placental function, inherited susceptibility, and postnatal conditions rather than as an isolated consequence of metformin.

Future studies should prespecify maternal indication, diabetes severity, glycemic trajectories, hypertensive and renal comorbidity, fetal growth status, metformin adherence and dose, supplemental insulin exposure, and major postnatal determinants. Analyses should separate the total effect of the treatment strategy from any hypothesized direct pharmacological effect and should use appropriate causal methods, including time-varying exposure models, active-comparator designs, propensity weighting, sibling comparisons where feasible, and sensitivity analyses for residual confounding. Until such evidence becomes available, causal attribution of long-term offspring outcomes directly to metformin should remain cautious.

#### 3.3.6. Long-Term Growth, Adiposity, and Cardiometabolic Outcomes

Evidence concerning long-term offspring growth and cardiometabolic health remains substantially less certain than evidence concerning maternal and neonatal outcomes. Available follow-up studies differ in maternal indication, treatment comparator, metformin dose and timing, geographic and ethnic setting, offspring age, participation rate, anthropometric method, and adjustment for maternal and postnatal confounders. Follow-up has generally extended only into infancy or mid-childhood, with very limited information during puberty and no sufficiently robust evidence concerning adult cardiometabolic disease.

The interpretation of childhood anthropometry depends strongly on the age at assessment. Earlier analyses of GDM trials suggested a pattern of lower birthweight followed by accelerated postnatal growth. In a 2019 meta-analysis, metformin-exposed offspring were approximately 440 g heavier at 18–24 months and had a BMI that was 0.78 kg/m^2^ higher at 5–9 years than offspring exposed to insulin [36]. However, these estimates were based on a small number of follow-up cohorts and should not be interpreted as evidence of a persistent adverse growth trajectory.

A more recent systematic review and meta-analysis included 18 studies reporting on 7975 metformin-exposed children and more than one million unexposed children [54]. At the oldest available assessment, corresponding to a weighted mean age of 4.4 years, BMI was comparable between exposed and unexposed children (standardized mean difference −0.02, 95% CI −0.11 to 0.07). Metformin-exposed children had a slightly higher BMI between one and three years of age (standardized mean difference 0.15, 95% CI 0.04–0.27), but this difference was no longer evident at ages three to six or six to eleven years. No overall differences were identified in the prevalence of overweight or obesity or in waist circumference. The certainty of this evidence was rated as low.

Findings differed according to maternal indication. In the same analysis, childhood BMI did not differ significantly in the diabetes or maternal-obesity subgroups, whereas children exposed to metformin in PCOS pregnancies were heavier than comparator-exposed children (standardized mean difference 0.31, 95% CI 0.00–0.62) [54]. This indication-specific pattern is consistent with the higher BMI reported in the PregMet offspring studies but argues against a uniform effect of metformin across all maternal metabolic conditions.

Several apparently conflicting long-term findings become more coherent when maternal indication, comparator treatment, offspring age, and the composition of the contributing cohorts are considered simultaneously. The 2019 meta-analysis restricted its primary comparison to pregnancies complicated by GDM and compared metformin-based treatment with insulin; it reported lower birthweight followed by greater weight at 18–24 months and a higher BMI at 5–9 years, although the childhood estimate was based on only 520 children from three studies [36]. By contrast, the more recent meta-analysis incorporated 18 studies across several maternal indications and different comparators and found no overall difference in BMI at the oldest available follow-up, while retaining a small increase at 1–3 years and an indication-specific signal in PCOS [54]. These findings are therefore not necessarily contradictory: the earlier signal may reflect age-specific catch-up growth within selected GDM cohorts, whereas the larger contemporary synthesis suggests that such differences are not consistently sustained across later childhood or across all maternal indications. The persistence of a higher BMI signal in PCOS pregnancies [46,47,54], but not in the diabetes or maternal-obesity subgroups, further argues against a uniform metformin-specific developmental effect and supports interaction with the underlying maternal metabolic and hormonal phenotype.

A similar indication- and comparator-dependent pattern is evident for SGA. In MiTy, which enrolled women with established pregestational T2DM receiving insulin, metformin increased the proportion of SGA infants from 7% to 13% [40]. Secondary analysis showed that both metformin exposure and pre-existing chronic hypertension and/or nephropathy independently predicted SGA, with the absolute risk reaching 25% among metformin-treated women with these vascular or renal comorbidities [42]. In contrast, the 2019 GDM meta-analysis found no significant difference in SGA between metformin- and insulin-treated pregnancies [36]. Comparator choice also altered the observational signal: in the Finnish register cohort, metformin was associated with higher SGA risk when insulin was the reference treatment (IPTW-weighted OR 1.65, 95% CI 1.16–2.34) [39], whereas no increased SGA risk was observed when metformin-exposed pregnancies were compared with non-pharmacologically treated GDM (weighted OR 1.01, 95% CI 0.75–1.37) [53]. Taken together, these findings suggest that the observed SGA signal is not uniform across pregnancy indications and may be amplified by baseline vascular or placental risk, diabetes severity, and the choice of comparator. They support particular caution in T2DM complicated by hypertension, nephropathy, or emerging placental insufficiency rather than assuming an equivalent fetal growth effect in uncomplicated GDM.

Individual follow-up cohorts further illustrate the heterogeneity. In MiG TOFU, metformin-exposed children had larger measures of subcutaneous fat at two years, but total fat mass and body-fat percentage were similar to those after insulin exposure [37]. At seven years, no anthropometric or metabolic differences were identified in the Adelaide cohort, whereas metformin-exposed children in Auckland were larger at nine years according to weight, waist circumference, and selected other measures [38]. Despite these differences in size, total and abdominal body-fat percentages and fasting metabolic biomarkers were similar. Follow-up participation differed markedly between centers, involving 109 of 181 eligible children in Adelaide and 99 of 396 in Auckland, creating a substantial possibility of selection bias.

Other GDM follow-up studies have not reproduced a consistent adverse pattern. In a Finnish randomized follow-up at nine years, anthropometric measures, BMI, waist-to-height ratio, and glucose metabolism were similar after metformin and insulin exposure, while selected lipid measurements were modestly more favorable after metformin. Observational growth-trajectory data from the Born in Bradford cohort also found comparable weight, height, and BMI trajectories from birth to 60 months between metformin- and insulin-exposed offspring. These findings suggest that differences identified in individual cohorts may depend on population characteristics, treatment practices, and follow-up participation rather than representing a universal developmental response.

In pregestational T2DM, MiTy Kids found no overall difference in BMI Z score, skinfold thickness, or growth trajectory at 24 months after metformin or placebo exposure [44]. However, only 61% of eligible children participated, and the exploratory finding of a different BMI trajectory among boys requires independent confirmation. Follow-up after two years is insufficient to evaluate later insulin resistance, dyslipidemia, hypertension, hepatic fat accumulation, or pubertal growth.

The PCOS follow-up studies reported a more consistent signal of higher childhood BMI, but their generalizability is limited. These studies were conducted predominantly in Norway, compared metformin with placebo rather than insulin, involved first-trimester-to-delivery exposure, and experienced substantial attrition. Consequently, the findings may reflect interactions between metformin, the PCOS intrauterine environment, maternal androgen and metabolic phenotype, shared familial susceptibility, and postnatal exposures. They should not be extrapolated automatically to pregnancies complicated by GDM or T2DM.

A 2024 meta-analysis focusing on offspring aged 5–11 years after maternal diabetes treatment found no significant differences between metformin and insulin in BMI, waist-to-height ratio, total fat mass, body-fat percentage, fat-free mass, visceral adipose tissue, liver fat, or major metabolic biomarkers [55]. Several estimates nevertheless had wide confidence intervals and substantial statistical heterogeneity, including heterogeneity in waist-to-height ratio, total fat mass, and visceral adipose tissue. In addition, the pooled results were derived from relatively few follow-up cohorts, several of which originated from the same parent trials and therefore did not represent fully independent populations.

Birthweight, childhood BMI, and adiposity should also be interpreted as related but non-interchangeable outcomes. A modestly lower birthweight followed by greater infant growth may indicate catch-up growth, but it does not necessarily predict later obesity or metabolic disease. Similarly, a small difference in childhood BMI does not establish increased visceral adiposity, insulin resistance, or future cardiovascular disease. Evaluation of developmental trajectories requires repeated measurements of growth, body composition, blood pressure, glucose metabolism, lipid profile, liver fat, and pubertal development rather than isolated anthropometric assessments.

The available evidence is therefore broadly reassuring but not definitive. Current studies do not demonstrate a consistent increase in childhood obesity, insulin resistance, diabetes, dyslipidemia, hypertension, or adverse body-fat distribution after prenatal metformin exposure. However, the absence of a statistically significant difference within relatively small and incomplete childhood cohorts does not establish long-term developmental neutrality. Clinically important effects emerging during puberty, pregnancy in female offspring, or adulthood remain insufficiently studied.

Overall, confidence is moderate that prenatal metformin exposure is not associated with a large difference in childhood BMI or adiposity across all maternal indications. Confidence is low regarding indication-specific growth effects, particularly in PCOS, and low regarding later cardiometabolic disease because of heterogeneous populations, inconsistent comparators, substantial attrition, limited ethnic diversity, and the absence of adequately powered follow-up into adulthood. The principal long-term studies and their sources of heterogeneity are summarized in Table 4.

#### 3.3.7. Neurodevelopmental, Immune, Reproductive, and Cardiovascular Outcomes

Long-term evidence outside anthropometric and metabolic outcomes remains comparatively limited. Neurodevelopmental, immune, reproductive, and cardiovascular outcomes have been assessed in fewer studies, frequently as secondary or exploratory endpoints. The available evidence also combines different maternal indications, treatment comparators, offspring ages, and outcome-assessment methods, limiting the certainty and generalizability of conclusions.

Current neurodevelopmental evidence is generally reassuring. A 2024 systematic review and meta-analysis included seven studies and 14,042 children, of whom 7641 had been exposed to metformin during pregnancy, with follow-up extending to 14 years of age [56]. Prenatal metformin exposure was not associated with neurodevelopmental delay during infancy (RR 1.09, 95% CI 0.54–2.17) or at three to five years of age (RR 0.90, 95% CI 0.56–1.45). Motor scores were also similar between exposed and comparator groups (mean difference 0.30, 95% CI −1.15 to 1.74), as were cognitive scores (mean difference −0.45, 95% CI −1.45 to 0.55).

These findings reduce concern regarding a large adverse effect on early cognitive or motor development. Nevertheless, most available studies used screening instruments, routinely recorded developmental diagnoses, or relatively broad composite outcomes. Evidence remains limited for executive function, attention, academic achievement, mental health, language complexity, and neurodevelopment after puberty. Differences in maternal glycemia, obesity, hypertensive disease, prematurity, and socioeconomic circumstances may also influence neurodevelopment independently of metformin exposure.

The Finnish register-based cohort found no increased risk of recorded motor-social developmental problems after metformin exposure compared with insulin [39]. Register-based findings are useful for identifying diagnosed disorders in large populations but may not detect subtle cognitive, behavioral, or educational differences. Accordingly, the absence of increased diagnostic codes should not be interpreted as proof that all neurodevelopmental domains are identical.

Evidence concerning immune and allergic outcomes is substantially less developed. A 2025 post hoc analysis of the PregMet, PregMet2, and PedMet cohorts included 145 offspring assessed at approximately eight years of age [57]. Prenatal metformin exposure in PCOS pregnancies was associated with higher reported odds of allergy (OR 4.83, 95% CI 1.47–21.8) and eczema (OR 2.42, 95% CI 1.14–5.33). The wide confidence intervals indicate considerable imprecision, and the analysis was based on a small, geographically restricted follow-up sample.

These immune findings should therefore be regarded as hypothesis-generating. They originated from post hoc analyses, involved multiple outcomes, and have not yet been replicated in an independent population or in pregnancies complicated by GDM or T2DM. Maternal PCOS-related immune dysregulation, familial atopy, environmental allergen exposure, antibiotic use, breastfeeding, childhood diet, and loss to follow-up may also have influenced the observed associations. The available evidence is insufficient to conclude that metformin causes childhood allergy or eczema.

Reproductive and endocrine outcomes have been examined primarily in offspring of women with PCOS. In a PregMet follow-up, 117 of 255 invited children aged five to ten years underwent steroid-hormone assessment [58]. Daughters of women with PCOS had higher androstenedione, testosterone, and calculated free-testosterone Z scores than a reference population. These differences were associated primarily with maternal PCOS status and cannot be attributed directly to metformin.

Among boys, prenatal metformin exposure was associated with a higher 11-deoxycortisol Z score than placebo exposure, with a mean difference of 0.65 (95% CI 0.14–1.17) [58]. However, the comparison involved only 31 metformin-exposed and 24 placebo-exposed boys, occurred before or during early pubertal development, and formed part of multiple hormonal analyses. Its clinical relevance remains uncertain. There is currently no robust human evidence demonstrating altered pubertal timing, testicular development, ovarian function, fertility, menstrual function, or reproductive capacity as a consequence of prenatal metformin exposure.

Cardiovascular evidence is also sparse. Childhood blood pressure, lipid concentrations, glucose metabolism, and measures of body composition were generally similar between metformin and comparator groups in the principal GDM follow-up studies. Some sex-specific differences in lipids, glucose, or adipokines have been reported, but these have not been consistent across cohorts and were commonly derived from secondary analyses. The Finnish register-based cohort did not identify an increased risk of diagnosed hypertension after metformin exposure [39].

No adequately powered studies have evaluated adult hypertension, vascular function, cardiac structure, atherosclerosis, cardiovascular events, or cardiovascular mortality after prenatal metformin exposure. Childhood blood pressure or lipid measurements cannot reliably establish adult cardiovascular safety or harm, particularly because later outcomes are strongly influenced by obesity, diet, physical activity, smoking, socioeconomic conditions, and the offspring’s own metabolic health.

Overall, confidence is moderate that prenatal metformin exposure is not associated with a large increase in early childhood neurodevelopmental delay. Confidence is low regarding immune and allergic outcomes and low or exploratory regarding reproductive, endocrine, and cardiovascular consequences. The reported allergy, eczema, and steroid-hormone findings require replication in larger, prospectively designed studies with prespecified outcomes, appropriate adjustment for maternal indication and familial risk, and follow-up through puberty and adulthood. The indication-specific balance between maternal benefit, fetal and neonatal outcomes, and long-term offspring uncertainty is summarized in Table 5.

Narrative confidence descriptors were assigned according to the GRADE-informed framework described in Section 2.6 and do not represent formal outcome-specific GRADE ratings. Maternal indications should not be compared as interchangeable exposure groups because they differ in disease severity, glycemic status, vascular and placental risk, treatment comparator, gestational timing of exposure, and baseline fetal growth phenotype. To make the sources of heterogeneity directly comparable, Table 6 presents a side-by-side appraisal of the principal discordant studies according to maternal indication, comparator treatment, geographic setting, offspring age or outcome timing, follow-up completeness, outcome definition, adjustment for confounding, and the principal methodological or biological factors that may explain divergent findings.

Taken together, these comparisons indicate that the apparent inconsistencies in fetal growth and childhood adiposity outcomes are not adequately explained by metformin exposure alone. The direction and magnitude of the observed association depend substantially on maternal indication, baseline metabolic and vascular risk, comparator treatment, gestational timing and duration of exposure, offspring age at assessment, follow-up completeness, outcome definition, and the extent to which maternal and postnatal confounding can be addressed. The Tarry-Adkins and Fu meta-analyses are therefore better interpreted as describing different portions of the postnatal growth trajectory rather than as mutually exclusive conclusions. Similarly, the PCOS-specific childhood BMI signal and the T2DM-specific SGA signal should not be generalized across all metformin-exposed pregnancies. These differences reinforce the indication-specific interpretation adopted throughout this review.

#### 3.3.8. Breastfeeding and Postnatal Metformin Exposure

Metformin is excreted into human milk, but the resulting infant exposure is substantially lower than fetal exposure occurring through placental transfer. In a pharmacokinetic study of seven breastfeeding women receiving a median metformin dose of 1500 mg/day, the mean milk-to-plasma concentration ratio was 0.35 (95% CI 0.20–0.50), and the mean metformin concentration in milk was 0.27 mg/L [59]. The estimated absolute infant dose was 0.04 mg/kg/day, while the mean relative infant dose was 0.28% of the maternal weight-adjusted dose (range 0.16–0.40%). Metformin concentrations were very low or undetectable in the plasma of the assessed infants, and no short-term health problems were identified.

A separate longitudinal pharmacokinetic study similarly estimated total infant intake through breast milk at approximately 0.13–0.28 mg/day, corresponding to a relative infant dose below 0.5% of the maternal weight-adjusted dose [8]. These values are markedly lower than umbilical cord concentrations at delivery, which may approach maternal plasma concentrations. Prenatal and postnatal exposure should therefore not be considered pharmacokinetically equivalent.

Limited clinical follow-up is also reassuring. Among infants born to women with PCOS who continued a median metformin dose of 2.55 g/day during lactation, growth, motor and social development, and the frequency of intercurrent illness did not differ between 61 breastfed infants and 50 formula-fed infants during the first six months of life [60]. However, this was not a randomized comparison of postnatal metformin exposure. All infants had already been exposed during pregnancy, feeding method was not assigned randomly, and the study was not powered to identify uncommon adverse effects or outcomes emerging after infancy.

The available evidence therefore supports low exposure through human milk and has not identified a short-term safety signal in healthy infants. Nevertheless, lactation evidence remains based on small pharmacokinetic samples and limited clinical follow-up. A 2026 systematic review of glucose-lowering medications during lactation found that only a small proportion of commonly used agents had direct human milk transfer data and that clinical recommendations remained inconsistent and were frequently not supported by direct clinical evidence [61].

Particular interpretive caution is appropriate for preterm infants, neonates with low birthweight, and infants with impaired renal function because metformin is eliminated predominantly by the kidneys and these populations were not adequately represented in the available studies. This does not establish an increased risk but identifies a population in which individualized clinical assessment and infant monitoring are reasonable.

Continued postpartum metformin treatment should be based primarily on the maternal indication, glycemic status, renal function, and expected clinical benefit. In a healthy term infant, the low relative infant dose alone does not provide a strong reason to discontinue either clinically indicated maternal metformin or breastfeeding. Routine interruption of breastfeeding or discarding expressed milk is not supported by the available pharmacokinetic evidence.

Breastfeeding should nevertheless be documented carefully in offspring follow-up studies. Duration and exclusivity of breastfeeding, maternal postpartum metformin dose, adherence, maternal glycemic control, introduction of complementary feeding, and infant growth may influence childhood metabolic outcomes. Failure to measure these variables may obscure whether postnatal growth differences are associated with prenatal exposure, additional exposure through milk, breastfeeding itself, or the broader maternal and family environment.

Overall, confidence is moderate that infant exposure to metformin through breast milk is very low and is not associated with an evident short-term adverse effect in healthy term infants. Confidence regarding preterm infants, infants with renal impairment, prolonged exposure, and health outcomes beyond early infancy remains low because the available studies are small and long-term lactation-specific follow-up is lacking.

## 4. Clinical Translation: Candidate Selection, Monitoring, and Shared Decision-Making

The clinical role of metformin during pregnancy cannot be defined by a single universal recommendation because the balance of benefit and uncertainty differs substantially among GDM, pregestational T2DM, PCOS, and maternal obesity without diabetes. Treatment decisions should therefore begin with confirmation of the maternal indication, assessment of disease severity and placental risk, and identification of the appropriate comparator treatment rather than with the general assumption that metformin is either uniformly beneficial or uniformly harmful.

In GDM, lifestyle and nutritional treatment remain the initial therapeutic approach, while insulin remains the preferred pharmacological treatment in major contemporary guidance when glycemic targets are not achieved [13]. Metformin may be considered in selected women after individualized counseling, particularly when insulin administration is not feasible, is declined after informed discussion, or would create substantial treatment-access or adherence barriers. However, treatment should not be delayed when hyperglycemia is marked or persistent, and women should be informed that supplemental insulin may still be required.

For practical treatment assessment, standard pregnancy glucose targets should remain the therapeutic reference irrespective of whether metformin or insulin is used: fasting glucose < 95 mg/dL (<5.3 mmol/L), 1 h postprandial glucose < 140 mg/dL (<7.8 mmol/L), and 2 h postprandial glucose < 120 mg/dL (<6.7 mmol/L) [13,14]. Persistent values above these targets despite adherence and appropriate dose titration should be regarded as inadequate glycemic control and should prompt timely treatment intensification rather than acceptance of less stringent targets because an oral medication is being used.

Appropriate candidates for a metformin-based GDM strategy are more likely to include women with moderate hyperglycemia, preserved renal function, no evidence of placental insufficiency or impaired fetal growth, and the ability to perform regular glucose monitoring. Insulin should be initiated or added promptly when fasting or postprandial glucose targets remain unmet, when hyperglycemia worsens with advancing gestation, or when there is evidence of significant fetal overgrowth despite treatment. Metformin treatment failure should be interpreted as insufficient glycemic efficacy rather than as a reason to tolerate prolonged suboptimal glucose control.

When metformin is selected for GDM, treatment may be initiated at 500 mg once daily with food to improve gastrointestinal tolerability. A practical trial-based strategy is then to increase the dose progressively according to glycemic response and tolerance. In SUGAR-DIP, metformin was initiated at 500 mg once daily and increased every 3 days to 1000 mg twice daily or the highest tolerated dose [34]. Other major pregnancy trials have used maximum daily doses of up to approximately 2500 mg [52]. Thus, a usual trial-based maintenance range is 1500–2000 mg/day in divided doses, with doses up to 2500 mg/day used in selected protocols when tolerated. These regimens should not be interpreted as a universally validated pregnancy-specific dosing algorithm. Failure to achieve pregnancy glucose targets at the maximum tolerated dose should prompt addition or transition to insulin rather than continued metformin escalation solely to avoid injectable treatment. In pregestational T2DM, insulin remains the principal treatment because of the greater degree of hyperglycemia, the need for rapid dose adjustment, and the high baseline risk of maternal vascular and fetal complications. The 2025 Endocrine Society and European Society of Endocrinology guideline suggested against the routine addition of metformin to insulin because the reduction in large-for-gestational-age birth was not considered to outweigh the possible increase in small-for-gestational-age birth and uncertainty regarding childhood body composition [14]. This recommendation was conditional and based on low-certainty evidence, but it argues against automatic continuation or initiation of metformin in every insulin-treated pregnancy.

An individualized adjunctive approach may still be discussed in selected women with marked insulin resistance, very high insulin requirements, excessive gestational weight gain, and fetal overgrowth, provided that maternal renal function is preserved and there is no evidence of impaired placental reserve. Particular caution is warranted in the presence of chronic hypertension, nephropathy, renal impairment, pre-eclampsia, reduced fetal growth velocity, suspected placental insufficiency, or established FGR. In these circumstances, the indication for continued metformin should be reassessed rather than assumed to remain unchanged throughout pregnancy.

In women with PCOS, the fact that metformin was used before conception does not in itself justify continuation until delivery. The original reason for treatment should be reviewed, including metabolic dysfunction, fertility treatment, previous pregnancy loss, or prevention of ovarian hyperstimulation. Randomized evidence does not support continuation solely to prevent GDM, and the possible reduction in late miscarriage or preterm birth should be balanced against uncertain long-term offspring growth findings. Decisions should distinguish preconception use, temporary continuation during early pregnancy, and prolonged exposure through later gestation.

Maternal overweight or obesity without diabetes is not an established indication for routine metformin treatment during pregnancy. Randomized trials have not demonstrated meaningful improvement in birthweight, major neonatal outcomes, or childhood obesity, despite modest reductions in gestational weight gain. Metformin should therefore not be initiated solely to restrict maternal weight gain or prevent obesity in the child. If GDM or overt T2DM subsequently develops, treatment should be reconsidered according to that metabolic diagnosis rather than obesity alone.

Before treatment initiation or continuation, maternal assessment should include the clinical indication, gestational age, glucose profile, current or anticipated insulin requirements, gastrointestinal tolerance, adherence, renal function, blood pressure, vascular and renal comorbidity, and concurrent medications. Metformin should be avoided or discontinued according to established non-pregnancy contraindications in the presence of clinically significant renal impairment, acute conditions associated with hypoxia or dehydration, or other circumstances increasing the risk of drug accumulation.

Maternal monitoring should include fasting and postprandial glucose measurements, treatment adherence, gastrointestinal adverse effects, weight trajectory, blood pressure, and renal function when clinically indicated. Maternal glucose monitoring should be interpreted against the pregnancy-specific glycemic targets defined above, with treatment intensified when these targets are not achieved consistently.

Fetal assessment should be based on the underlying maternal disease and obstetric risk rather than on metformin exposure alone. Serial assessment of fetal biometry and growth velocity is particularly relevant in women with T2DM, chronic hypertension, nephropathy, previous FGR, or other conditions associated with placental dysfunction. When estimated fetal weight or abdominal circumference falls below the 10th percentile, fetal growth velocity declines, maternal hypertensive disease develops, or other signs of placental dysfunction emerge, fetal surveillance should be intensified according to established FGR guidance. Emerging FGR, abnormal umbilical artery Doppler findings, or other evidence of placental insufficiency should trigger active reassessment of the indication for continued metformin, particularly in women with T2DM and coexisting hypertension or nephropathy. Importantly, no metformin-specific fetal biometric percentile, growth-velocity threshold, umbilical artery Doppler criterion, or other surveillance parameter has been validated as an evidence-based automatic stopping rule, and metformin dose reduction has not been shown to prevent SGA or FGR. Therefore, fetal growth or Doppler abnormalities should prompt individualized benefit–risk reassessment and management according to established FGR principles rather than automatic discontinuation on the basis of an unvalidated drug-specific threshold.

Shared decision-making should include an explicit discussion of both established evidence and unresolved uncertainty. A practical counseling sequence should address four issues: first, why pharmacological treatment is required and the maternal and fetal consequences of persistent hyperglycemia; second, the expected short-term advantages and disadvantages of metformin compared with insulin, including oral versus injectable administration, gastrointestinal adverse effects, maternal hypoglycemia, treatment burden, cost and availability, gestational weight gain, and the possible need for supplemental insulin; third, the fact that metformin crosses the placenta and that current evidence has not demonstrated a consistent major short-term safety signal, while long-term offspring evidence remains incomplete; and fourth, the circumstances that would prompt treatment escalation or reconsideration, including failure to achieve glycemic targets, development of a maternal contraindication, or emerging fetal growth or placental concerns. Counseling should avoid presenting metformin as equivalent to insulin in every clinical context but should equally avoid presenting placental transfer alone as evidence of developmental harm. Patient preferences are clinically relevant but should be elicited after balanced information has been provided. In a study of 144 women with GDM requiring pharmacological therapy, women receiving metformin more frequently prioritized avoiding injections and limiting medication frequency, whereas women receiving insulin more frequently prioritized avoiding a medication that crosses the placenta and avoiding the possibility of later supplemental therapy [62]. Decisional comfort and satisfaction were high with both treatments, suggesting that patient-centered counseling can support acceptable decisions even when preferences differ.

**Figure 4:**
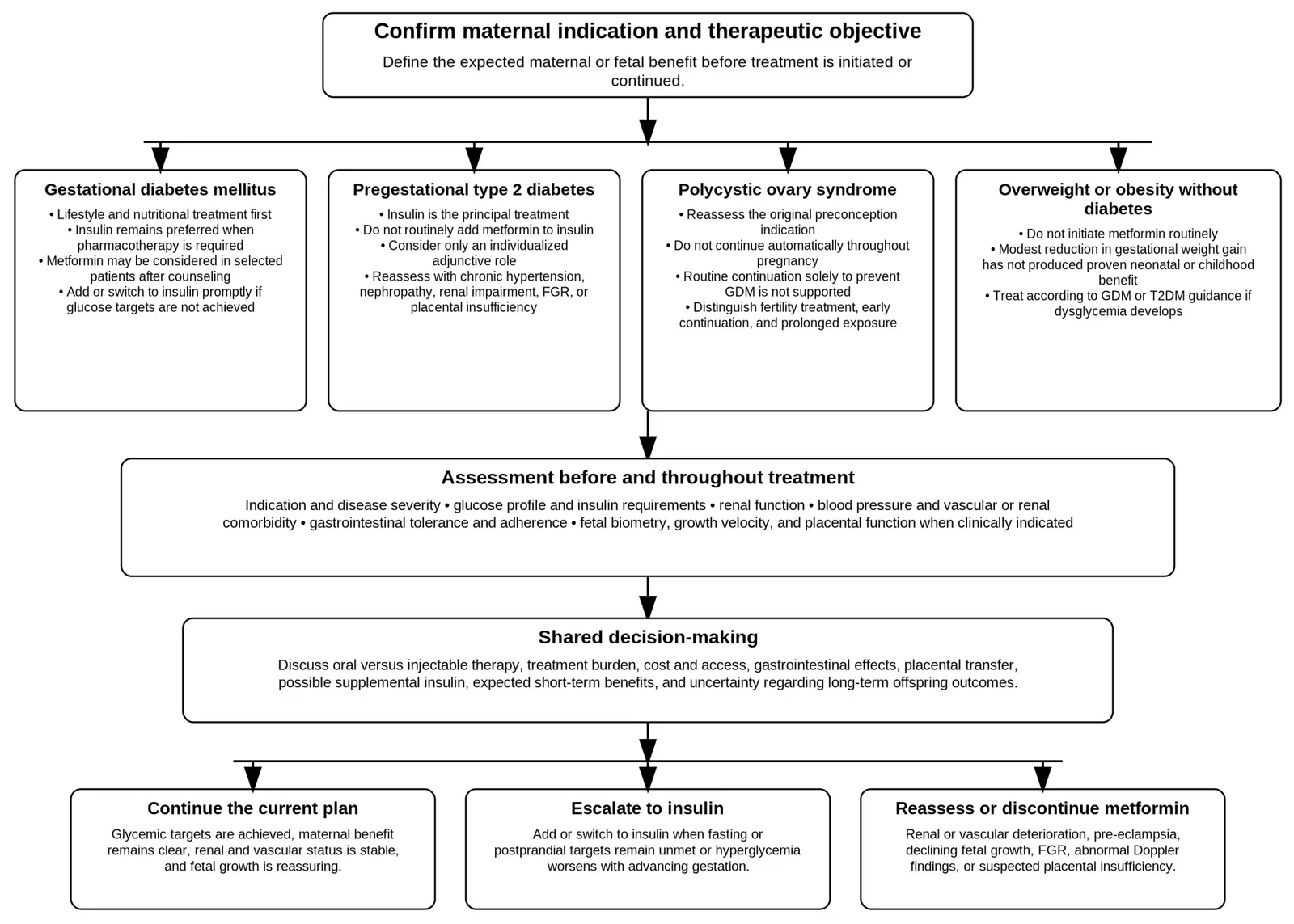
Indication-specific clinical decision pathway for metformin use during pregnancy. Treatment decisions should begin with confirmation of the maternal indication and the expected therapeutic benefit. Gestational diabetes mellitus, pregestational type 2 diabetes mellitus, polycystic ovary syndrome, and maternal overweight or obesity without diabetes represent distinct clinical contexts and should not be managed using a single universal approach. Across indications, treatment should be reassessed according to glycemic response, maternal renal and vascular status, treatment tolerability, fetal growth, and placental function. Metformin should not delay insulin initiation or escalation when glycemic targets are not achieved. Emerging fetal growth restriction, abnormal Doppler findings, preeclampsia, renal deterioration, or suspected placental insufficiency should prompt reassessment of the benefit–risk balance.

Shared decision-making does not remove the clinician’s responsibility to recommend insulin when it is more likely to achieve safe and timely glycemic control. Conversely, a preference for oral treatment should not be dismissed automatically when metformin represents a clinically reasonable option. The final treatment decision should be documented together with the maternal indication, alternatives discussed, the patient’s treatment priorities, acknowledgment of placental transfer and uncertainty regarding long-term offspring evidence, planned glucose monitoring, criteria for insulin initiation or escalation, relevant maternal contraindications, and the fetal growth surveillance strategy. Overall, the most appropriate clinical approach is an indication-specific and dynamically reassessed treatment pathway. Metformin may provide meaningful maternal and short-term neonatal benefits in selected pregnancies, but its use should be linked to a defined therapeutic objective, regular evaluation of glycemic response, maternal renal and vascular status, and fetal growth. An indication-specific clinical decision pathway is summarized in Figure 4.

## 5. Discussion

This focused narrative review demonstrates that the maternal, fetal, and offspring effects of metformin during pregnancy cannot be summarized by a single conclusion that is applicable across all clinical indications. The evidence is most robust for selected short-term maternal and neonatal outcomes, whereas certainty decreases substantially for direct placental mechanisms, childhood growth trajectories, and health outcomes emerging after puberty. The balance between benefit and uncertainty therefore depends on the maternal indication, disease severity, treatment comparator, gestational timing, maternal renal and vascular status, glycemic response, placental function, and fetal growth pattern. Across the available evidence, maternal indication and metabolic severity are not merely background characteristics but central determinants of treatment selection, placental function, fetal growth, and postnatal risk; they therefore limit any simple attribution of offspring outcomes to metformin exposure alone.

In GDM, randomized trials and meta-analyses generally support comparable glycemic efficacy between metformin-based treatment and insulin, together with less maternal gestational weight gain, a lower risk of neonatal hypoglycemia, and modestly lower birthweight [33,34,35,36,37,38,39,52]. However, a considerable proportion of women require supplemental insulin, and metformin should therefore not be presented as reliable monotherapy for every patient. The reduction in neonatal hypoglycemia and excessive fetal growth represents a clinically relevant short-term benefit, but lower mean birthweight should not automatically be interpreted as improved fetal development. Available GDM evidence does not demonstrate a consistent increase in SGA, although differences among comparators, treatment timing, populations, and definitions remain important.

Pregestational T2DM represents a distinct and higher-risk clinical context. The MiTy and MOMPOD trials indicate that adding metformin to insulin can reduce insulin requirements, maternal gestational weight gain, birthweight, and the probability of extreme fetal overgrowth without improving broad composite neonatal outcomes [40,43]. The increased SGA signal observed in MiTy, particularly among women with chronic hypertension or nephropathy, changes the clinical interpretation from one of general metabolic benefit to one of potential growth trade-off. The reduction in LGA and the increase in SGA should therefore be considered together rather than reported as unrelated outcomes.

This distinction also explains why contemporary guidance advises against the routine addition of metformin to insulin in every pregnancy complicated by T2DM. The recommendation does not establish that metformin is universally harmful in T2DM pregnancy, but it indicates that expected maternal benefits may not outweigh potential fetal growth concerns in all patients. Women with marked insulin resistance and excessive fetal growth may differ substantially from women with nephropathy, chronic hypertension, impaired placental reserve, or declining fetal growth velocity. A dynamically reassessed, phenotype-specific approach is therefore more appropriate than either routine addition or universal avoidance.

In PCOS, evidence should be interpreted according to the original reason for metformin treatment and the duration of pregnancy exposure. PregMet2 did not demonstrate a statistically significant reduction in its primary composite outcome, although a post hoc pooled analysis suggested a possible reduction in late miscarriage or preterm birth [45]. Metformin did not prevent GDM, and routine continuation solely for that purpose is not supported. The finding of higher childhood BMI in the PregMet and PedMet follow-up studies is clinically relevant but remains limited by sample size, attrition, geographic concentration, and uncertainty regarding whether the observed phenotype reflects metformin, maternal PCOS, familial metabolic susceptibility, or their interaction [46,47].

Maternal overweight or obesity without diabetes is the indication for which the evidence is least supportive of routine treatment. EMPOWaR, the severe-obesity trial, and GRoW did not demonstrate meaningful improvement in neonatal birthweight, major pregnancy outcomes, or childhood obesity, despite modest reductions in gestational weight gain [48,49,50]. These findings illustrate that a change in an intermediate maternal outcome does not necessarily translate into improved fetal or childhood health. Maternal obesity alone should therefore not be regarded as an indication for pharmacological fetal exposure.

The biological interpretation is similarly indication-dependent. Improvement in maternal glycemia, insulin resistance, gestational weight gain, and systemic metabolic stress represents the most clinically established pathway through which metformin may influence pregnancy outcomes. By contrast, direct effects on placental mitochondrial respiration, AMPK–mTOR signaling, nutrient transport, epigenetic regulation, or fetal cellular metabolism remain based predominantly on placental explants, isolated trophoblasts, animal models, and other experimental systems. These findings support biological plausibility but do not establish that a clinically meaningful developmental effect occurs in human offspring.

Placental transfer should therefore be interpreted neither as proof of developmental toxicity nor as evidence of biological neutrality. Fetal concentrations may approach maternal concentrations, but the net developmental effect reflects both direct drug exposure and reduction in exposure to maternal hyperglycemia, hyperinsulinemia, inflammation, excessive nutrient availability, and other consequences of metabolic disease. The relevant clinical comparison is not metformin exposure versus an uncomplicated metabolically healthy pregnancy, but metformin-based treatment versus insulin, placebo, lifestyle treatment, or no pharmacological therapy within a clearly defined maternal indication.

Long-term offspring evidence is broadly reassuring but remains insufficient for definitive conclusions. Recent meta-analytic evidence found no overall difference in childhood BMI at the oldest available follow-up, although a small increase was observed at one to three years and indication-specific differences were identified in PCOS cohorts [54]. Studies involving GDM and T2DM have generally not demonstrated consistent differences in childhood body-fat percentage, glucose metabolism, blood pressure, or lipid profile. Neurodevelopmental evidence is also reassuring with respect to large effects on early cognitive and motor development. However, these findings cannot establish developmental neutrality because most follow-up remains limited to infancy or mid-childhood, participation is incomplete, and adequately powered data after puberty are absent.

Findings concerning childhood allergy, eczema, steroid hormones, sex-specific growth trajectories, and selected metabolic biomarkers should remain exploratory. They were generally derived from post hoc analyses, relatively small subgroups, multiple comparisons, or single geographic cohorts. Such findings are important for hypothesis generation and the design of future follow-up studies, but they should not currently determine treatment independently of the established maternal indication and pregnancy-specific risk profile.

Confounding by indication remains a major limitation of observational evidence. Women treated with metformin may have either more severe disease than those managed by lifestyle treatment or less severe disease than those requiring insulin. Supplemental insulin, treatment discontinuation, renal and vascular comorbidity, maternal obesity, and clinician treatment preferences may further influence observed associations. Even randomized offspring follow-up studies remain vulnerable to differential attrition and to postnatal influences, including breastfeeding, parental adiposity, infant feeding, childhood diet, physical activity, socioeconomic circumstances, and shared genetic susceptibility.

From a clinical perspective, the evidence supports neither unrestricted metformin use nor categorical avoidance. Metformin may reduce treatment burden, maternal weight gain, neonatal hypoglycemia, and excessive fetal growth in selected pregnancies. These potential advantages should be balanced against gastrointestinal intolerance, placental transfer, possible need for supplemental insulin, the T2DM-specific SGA signal, and uncertainty concerning later offspring outcomes. The indication-specific pathway presented in Figure 4 translates this evidence into candidate selection, monitoring, insulin-escalation criteria, and shared decision-making rather than proposing a rigid universal protocol.

Patient preferences are an essential component of this process. Some women prioritize oral administration, avoidance of injections, lower cost, and reduced dosing burden, whereas others place greater importance on avoiding a medication that crosses the placenta or on choosing a treatment with longer-established pregnancy-specific experience. Balanced counseling should clarify that metformin and insulin differ in administration, placental transfer, maternal effects, and the certainty of long-term evidence. Shared decision-making should support informed choice without delaying insulin when timely and reliable glycemic control requires it.

### 5.1. Strengths and Limitations

A principal strength of this review is the indication-specific synthesis of evidence from GDM, pregestational T2DM, PCOS, and maternal obesity without diabetes. Human randomized and observational evidence was separated from placental, animal, and in vitro findings, and mechanistic plausibility was not treated as proof of clinical causality. The review also incorporated dose, timing, placental transfer, maternal renal function, supplemental insulin, fetal growth outcomes, breastfeeding exposure, effect estimates with 95% confidence intervals, and contemporary clinical guidance. Long-term evidence was evaluated according to offspring age, comparator treatment, geographic population, follow-up participation, outcome measurement, and maternal indication.

Several limitations should nevertheless be acknowledged. This was a focused narrative review rather than a systematic review or meta-analysis. The literature identification was targeted and iterative, and formal PRISMA record counts were not prospectively recorded. Although predefined eligibility domains, structured thematic organization, and narrative confidence descriptors were used to improve transparency, no validated study-level risk-of-bias instrument or formal GRADE assessment was applied. The review may therefore remain vulnerable to selection and interpretation bias.

The search prioritized the English-language and peer-reviewed literature, which may have excluded relevant evidence published in other languages or available through regulatory and unpublished sources. Conference abstracts were considered only selectively, and publication bias could not be assessed formally. Furthermore, several clinical outcomes were reported inconsistently across trials, and numerical pooling was not undertaken because maternal indications, comparators, exposure timing, supplemental therapy, and outcome definitions differed substantially.

The primary evidence also has important limitations. Many offspring studies represent secondary follow-up of trials originally powered for maternal or neonatal rather than childhood outcomes. Follow-up rates were frequently low, differed between treatment groups or geographic centers, and declined with increasing child age. Some publications arose from the same parent trials and therefore do not represent independent populations. Evidence concerning immune, reproductive, endocrine, and cardiovascular outcomes remains particularly sparse and is often based on exploratory analyses.

Mechanistic studies were heterogeneous in gestational timing, tissue source, experimental concentration, fetal sex, and model system. Findings from term placental tissue may not reflect effects during implantation, organogenesis, or earlier placental development. Experimental concentrations sometimes exceeded maternal concentrations achieved during clinical treatment, and direct relationships among maternal dose, placental concentration, fetal exposure, molecular response, and childhood phenotype remain insufficiently characterized. Additional heterogeneity arose from differences in diagnostic criteria, glycemic treatment targets, metformin dose and gestational timing, criteria for supplemental insulin, outcome definitions, and duration of offspring follow-up. These differences limited direct comparison among studies and may have contributed to apparently conflicting findings across maternal indications and offspring age groups.

### 5.2. Research Priorities

Future research should move from general comparisons of metformin exposure versus non-exposure toward indication-specific and phenotype-specific evaluation. Randomized and pragmatic comparative-effectiveness trials should report maternal metabolic phenotype, baseline glycemic severity, renal and vascular comorbidity, placental risk, fetal growth pattern, treatment adherence, gastrointestinal intolerance, supplemental insulin, and reasons for treatment escalation or discontinuation.

Pharmacokinetic studies should incorporate metformin dose, formulation, gestational timing, maternal renal function, serial maternal concentrations, cord concentrations, placental transporter expression, and, where feasible, transporter genotype. These data are required to determine whether prescribed dose, maternal systemic exposure, and fetal exposure are sufficiently correlated to support meaningful dose–response analysis.

Fetal growth outcomes should distinguish birthweight-based SGA from antenatally diagnosed FGR. Future trials should report longitudinal estimated fetal weight and abdominal circumference, growth velocity, placental and umbilical artery Doppler findings, hypertensive disease, gestational age at delivery, and neonatal phenotype. This would clarify whether lower birthweight represents reduction in pathological overgrowth, constitutional smallness, or placental growth restriction.

Offspring follow-up should extend through puberty and adulthood and include repeated assessment of growth, body composition, visceral and hepatic fat, glucose metabolism, lipid profile, blood pressure, vascular function, neurodevelopment, mental health, immune and allergic disease, pubertal timing, reproductive function, and cardiovascular outcomes. Breastfeeding duration, postpartum metformin exposure, complementary feeding, childhood diet, physical activity, parental adiposity, and socioeconomic conditions should be collected prospectively.

Greater geographic and ethnic diversity is also required. Many existing randomized follow-up cohorts originate from a limited number of high-income settings, and their findings may not be transferable to populations differing in diabetes phenotype, nutritional environment, body composition, healthcare access, and treatment practices. Individual-participant-data meta-analyses, harmonized core outcome sets, pregnancy registries, linkage with childhood health data, and causal-inference methods may improve precision and help identify subgroups in whom maternal benefit is most likely to outweigh fetal or offspring uncertainty.

## 6. Conclusions

Metformin is a clinically useful treatment in selected pregnancies, but its benefit–risk profile is not uniform across maternal indications. The strongest evidence concerns short-term maternal and neonatal outcomes, whereas certainty is substantially lower for direct placental and fetal mechanisms and for health outcomes extending beyond childhood. Clinical interpretation should therefore remain specific to the maternal diagnosis, treatment comparator, metabolic phenotype, gestational timing, renal and vascular status, placental function, and fetal growth trajectory.

In GDM, metformin-based treatment may reduce maternal gestational weight gain, neonatal hypoglycemia, and excessive fetal growth, although supplemental insulin is frequently required. In pregestational T2DM, adjunctive metformin may reduce insulin requirements and large-for-gestational-age birth, but the increased SGA signal—particularly among women with chronic hypertension or nephropathy—argues against routine addition to insulin in all pregnancies. In PCOS, possible effects on late miscarriage or preterm birth remain uncertain, and continuation throughout pregnancy solely to prevent GDM is not supported. Maternal overweight or obesity without diabetes is not an established indication for routine metformin treatment during pregnancy.

Metformin crosses the placenta and produces substantial fetal exposure. Experimental studies demonstrate biologically plausible effects on placental mitochondrial function, AMPK–mTOR signaling, nutrient sensing, and epigenetic regulation. However, these findings do not establish that metformin causes either beneficial or adverse developmental programming in human offspring. The net fetal effect cannot be separated from the concurrent reduction in exposure to maternal hyperglycemia, insulin resistance, excessive nutrient availability, and inflammation.

Available childhood follow-up is broadly reassuring. Current evidence does not demonstrate a consistent increase in childhood obesity, impaired glucose metabolism, hypertension, adverse body-fat distribution, or neurodevelopmental delay. Nevertheless, the evidence is limited by heterogeneous maternal indications and comparators, relatively small follow-up cohorts, substantial attrition, inconsistent outcome assessment, and insufficient control of postnatal confounders. No adequately powered evidence is available regarding outcomes after puberty, reproductive health, adult cardiometabolic disease, or cardiovascular events. Consequently, the absence of a demonstrated adverse effect should not be interpreted as proof of lifelong developmental neutrality [54,55,56].

Reported childhood phenotypes cannot presently be separated with confidence from the effects of maternal obesity, diabetes severity, vascular and placental dysfunction, supplemental insulin, inherited susceptibility, and the shared postnatal environment.

Metformin should therefore neither be adopted universally nor avoided categorically. Its use should be linked to a clearly defined maternal therapeutic objective and accompanied by regular assessment of glycemic response, need for insulin escalation, renal function, vascular and hypertensive comorbidity, treatment tolerability, and fetal growth. Particular caution is appropriate when placental insufficiency, declining fetal growth velocity, FGR, chronic hypertension, nephropathy, or impaired renal function is present or develops during treatment.

Shared decision-making should include the practical advantages of oral treatment, lower treatment burden, and selected short-term maternal and neonatal benefits, together with placental transfer, gastrointestinal adverse effects, possible need for supplemental insulin, the T2DM-specific SGA signal, and uncertainty regarding long-term offspring health. On the basis of current evidence, metformin represents a reasonable option for carefully selected patients when the expected maternal and pregnancy-specific benefit is judged to outweigh the remaining fetal and long-term uncertainty. Continued follow-up of exposed offspring into adolescence and adulthood remains essential.

## Figures and Tables

**Table 1:** Pharmacological characteristics of metformin relevant to fetal programming.

Characteristic	Maternal Relevance	Placental/Fetal Relevance	Programming Interpretation
Primary metabolic action	Reduces hepatic glucose production and improves insulin sensitivity	Reduces fetal exposure to maternal hyperglycemia and hyperinsulinemia	Potential indirect benefit through metabolic normalization
Cellular pathways	Influences mitochondrial activity, AMPK, inflammation, and nutrient sensing	The same pathways participate in placental transport and fetal growth	Creates biological plausibility for direct developmental effects
Placental transfer	Oral therapy with systemic maternal exposure	Readily crosses the placenta; fetal levels may approach maternal levels	Requires interpretation beyond maternal efficacy alone
Timing	May begin before conception or during any trimester	Different windows overlap with placentation, organogenesis, and rapid fetal growth	Exposure window should be reported explicitly
Growth effects	May reduce excessive gestational weight gain and fetal overgrowth	Lower birthweight or SGA may occur in selected populations	Benefit depends on maternal phenotype and placental reserve
Comparator	Often compared with insulin, placebo, or no treatment	Insulin does not cross the placenta significantly	Comparator choice strongly affects developmental interpretation

**Table 2:** Maternal–placental–fetal pathways potentially associated with metformin exposure and certainty of the supporting evidence.

Pathway or Domain	Effect of Maternal Metabolic Disease	Potential Metformin-Related Effect	Predominant Evidence Source and Narrative Confidence	Interpretation for Fetal or Offspring Outcomes
Maternal glycemia and insulin resistance	Maternal hyperglycemia increases fetal glucose exposure, fetal hyperinsulinemia, excessive nutrient availability, and the risk of fetal overgrowth.	Improves glycemic control in responsive patients and may reduce maternal insulin resistance, gestational weight gain, and the requirement for supplemental insulin.	Human randomized trials and meta-analyses; high-to-moderate confidence, depending on maternal indication and comparator.	May reduce large-for-gestational-age birth, macrosomia, and neonatal hypoglycemia. The magnitude of benefit differs between GDM and pregestational T2DM.
Placental nutrient transport	Diabetes and obesity may dysregulate placental glucose, amino acid, and lipid transport and may contribute to fetal overgrowth or, in the presence of placental insufficiency, impaired fetal growth [5,6].	Indirect normalization may occur through improved maternal metabolism. Direct effects on placental transporters and nutrient allocation remain proposed rather than clinically established.	Human placental observational studies and experimental models; low confidence for a direct metformin-specific effect.	Altered nutrient transport provides a plausible link with differences in birthweight and postnatal growth, but clinical causality has not been demonstrated.
Placental mitochondrial function	Maternal diabetes and obesity are associated with oxidative stress, altered mitochondrial biogenesis, impaired oxidative phosphorylation, and lipid accumulation.	In primary human trophoblasts, metformin reduced mitochondrial respiration and ATP production while also reducing markers of oxidative stress [23].	Human primary trophoblast and placental explant studies; low confidence for clinical outcomes.	The net in vivo effect may be adaptive, neutral, or adverse depending on placental metabolic stress, reserve, dose, and timing.
AMPK–mTOR and nutrient-sensing pathways	Excessive insulin, nutrient, and growth-factor signaling may activate placental mTOR and increase anabolic activity and nutrient transport.	Metformin may activate AMPK and attenuate mTOR-related growth signaling [25,26].	Predominantly animal and in vitro evidence; low or exploratory confidence.	Reduced anabolic signaling may be beneficial in fetal overgrowth but could be undesirable in pregnancies with impaired placental reserve or established risk of fetal growth restriction.
Inflammation, oxidative stress, and vascular function	Diabetes and obesity are associated with chronic inflammation, endothelial dysfunction, oxidative stress, and altered placental perfusion.	Improved maternal metabolism may indirectly reduce inflammatory and oxidative stress. Direct placental anti-inflammatory and vascular effects remain incompletely defined.	Human clinical evidence for indirect maternal effects and experimental evidence for direct placental effects; moderate confidence for maternal effects and low confidence for direct fetal effects.	Potential effects on placental function and fetal growth are biologically plausible but cannot be separated reliably from improvement in maternal metabolic disease.
Epigenetic regulation and mitochondrial biogenesis	Maternal diabetes is associated with changes in placental DNA methylation, histone modification, and transcriptional regulation.	Limited placental explant and animal studies suggest AMPK-dependent changes in PGC-1α expression, histone acetylation, and promoter methylation [26].	Small human placental explant studies and animal models; low or exploratory confidence.	Evidence supports biological plausibility but does not establish a persistent metformin-specific epigenetic signature or a causal long-term offspring effect.
Placental aging and senescence	Accelerated placental aging may occur in pregnancies complicated by fetal growth restriction, pre-eclampsia, or other placental disorders.	In randomized human placental samples, metformin was not associated with changes in telomere length, gene expression, DNA methylation, fibrosis, calcification, or other aging markers [27].	Human placental samples from a randomized trial with complementary experimental models; moderate confidence for the absence of a major aging-related effect in the studied population.	Available evidence does not support either acceleration or deceleration of placental aging by metformin, but generalization to other maternal indications remains limited.
Fetal sex-specific placental responses	Male and female placentas may differ in growth strategy, mitochondrial adaptation, inflammatory signaling, and susceptibility to maternal metabolic stress.	Sexually dimorphic mitochondrial and oxidative responses to metformin have been reported in human trophoblast experiments [24].	Small human placental and in vitro studies; exploratory confidence.	Sex-specific effects remain hypothesis-generating. Clinical trials and offspring follow-up studies require prespecified sex-stratified analyses.
Postnatal growth environment	Parental adiposity, infant feeding, diet, physical activity, socioeconomic circumstances, and catch-up growth influence the childhood phenotype.	Prenatal metformin treatment does not directly control these postnatal exposures. Postnatal exposure through breastfeeding is considered separately.	Observational evidence concerning postnatal determinants; not evidence of a direct metformin effect.	These factors are essential confounders and mediators in long-term offspring studies and may amplify, attenuate, or obscure associations with prenatal treatment.

**Table 3:** Principal randomized trials of metformin during pregnancy and their relevance to maternal, fetal, and developmental interpretation.

Trial	Maternal Indication and Design	Sample Size	Intervention and Comparator	Principal Findings	Main Limitations and Interpretative Considerations
MiG [33]	Multicenter, open-label randomized trial in women with GDM requiring pharmacological treatment at 20–33 weeks of gestation	751	Metformin, with supplemental insulin when required, versus insulin	The primary composite neonatal outcome occurred in 32.0% of the metformin group and 32.2% of the insulin group (RR 0.99, 95% CI 0.80–1.23). Supplemental insulin was required in 46.3% of women assigned to metformin. Treatment acceptability was greater with metformin, with 76.6% versus 27.2% of participants indicating that they would choose the assigned treatment again.	Open-label design; nearly half of the metformin group received supplemental insulin; treatment was initiated predominantly in the second or third trimester; the trial was powered for short-term neonatal morbidity rather than long-term developmental outcomes.
EMERGE [52]	Double-blind, placebo-controlled randomized trial evaluating early metformin in women with GDM diagnosed according to WHO 2013 criteria	535 pregnancies in 510 participants	Metformin up to 2500 mg/day plus usual care versus placebo plus usual care	The primary composite of insulin initiation or fasting glucose ≥ 5.1 mmol/L at 32 or 38 weeks was not significantly reduced: 56.8% versus 63.7% (RR 0.89, 95% CI 0.78–1.02). Metformin delayed insulin initiation, reduced gestational weight gain, and was associated with smaller neonatal anthropometric measures, without reduction in major neonatal morbidity.	The primary outcome was metabolic and treatment-related rather than a direct neonatal endpoint; conducted in two Irish centers; several favorable maternal and neonatal findings were secondary outcomes; long-term offspring follow-up is not yet available.
SUGAR-DIP [34]	Multicenter, open-label, noninferiority randomized trial in women with GDM inadequately controlled by diet	820	Sequential oral strategy beginning with metformin, followed when necessary by glyburide and later insulin, versus insulin-based treatment	The oral-agent strategy did not meet the prespecified noninferiority criterion for LGA: 23.9% versus 19.9%, absolute risk difference 4.0% (95% CI −1.7 to 9.8). Overall, 79% of participants assigned to the oral strategy maintained glycemic control without insulin.	This was not a trial of metformin monotherapy because glyburide and insulin could be added sequentially. The open-label design and treatment-strategy comparison prevent attribution of all findings specifically to metformin. Long-term offspring outcomes were not assessed.
MiTy [40]	International, multicenter, double-blind, placebo-controlled randomized trial in women with pregestational T2DM receiving insulin	502	Metformin 1000 mg twice daily plus insulin versus placebo plus insulin	The primary composite neonatal outcome was identical in both groups at 40% (RR 1.02, 95% CI 0.83–1.26). Metformin improved glycemic control, reduced insulin dose and gestational weight gain, and reduced cesarean birth and excessive fetal growth. Mean birthweight was 218 g lower, but SGA occurred in 13% versus 7% (RR 1.96, 95% CI 1.10–3.64).	Metformin was used only as an adjunct to insulin. Reduced LGA and increased SGA represent a clinically important fetal growth trade-off. The population had established T2DM and cannot be combined directly with GDM or PCOS cohorts.
MOMPOD [43]	Multicenter, double-blind randomized trial in women with preexisting T2DM or diabetes diagnosed before 23 weeks who were receiving insulin	831 randomized; 794 included in the primary analysis	Metformin 1000 mg twice daily plus insulin versus placebo plus insulin	The composite adverse neonatal outcome occurred in 71% versus 74% (adjusted OR 0.86, 95% CI 0.63–1.19). The trial was stopped at 75% of planned accrual for futility. Metformin reduced the odds of LGA birth (adjusted OR 0.63, 95% CI 0.46–0.86) but did not improve the broad neonatal composite.	The population combined preexisting T2DM and diabetes diagnosed early in pregnancy. Early stopping reduced statistical power for less frequent outcomes. Reduction in LGA was a component-level secondary finding rather than evidence of improvement in overall neonatal morbidity.
PregMet2 [45]	Multicenter, double-blind, placebo-controlled randomized trial in pregnant women with PCOS	487	Metformin 500 mg twice daily during the first week, increased to 1000 mg twice daily until delivery, versus placebo	The primary composite of late miscarriage or preterm birth occurred in 5% versus 10% (OR 0.50, 95% CI 0.22–1.08; *p* = 0.08) and was not statistically significant. In a post hoc pooled analysis with two earlier trials, the OR was 0.43 (95% CI 0.23–0.79). GDM was not prevented: 25% versus 24% (OR 1.09, 95% CI 0.69–1.66).	The principal trial did not reach statistical significance for its primary outcome. The significant result arose from a post hoc pooled analysis. PCOS phenotype, fertility treatment, obesity, insulin resistance, and hyperandrogenism may independently influence pregnancy and offspring outcomes.
EMPOWaR [48]	Multicenter, double-blind, placebo-controlled randomized trial in women with obesity and normal glucose tolerance	449 randomized; 434 included in the modified intention-to-treat analysis	Metformin titrated to a maximum of 2500 mg/day from 12 to 16 weeks until delivery versus placebo	Mean birthweight was almost identical: 3462 versus 3463 g. There was no significant effect on the standardized birthweight outcome (adjusted mean difference −0.029, 95% CI −0.217 to 0.158).	Maternal obesity without diabetes is not equivalent to a hyperglycemic treatment indication. The trial did not demonstrate fetal benefit, and it was not designed to establish long-term developmental safety.
Syngelaki et al. [49]	Double-blind, placebo-controlled randomized trial in women without diabetes and BMI >35 kg/m^2^	450 randomized; 400 included after withdrawals	Metformin 3 g/day from 12 to 18 weeks until delivery versus placebo	Metformin did not significantly reduce neonatal birthweight Z score. Maternal gestational weight gain was lower: 4.6 versus 6.3 kg. Pre-eclampsia occurred in 3.0% versus 11.3% (OR 0.24, 95% CI 0.10–0.61), but other trials have not consistently replicated this effect.	The pre-eclampsia finding was a secondary outcome and should not be interpreted as definitive evidence of prevention. Gastrointestinal adverse effects were more frequent. No clear fetal or neonatal benefit was demonstrated.
GRoW [50]	Multicenter, double-blind, placebo-controlled randomized trial in pregnant women with BMI ≥ 25 kg/m^2^ receiving dietary and lifestyle intervention	524 randomized; 514 included in outcome analyses	Metformin up to 2000 mg/day plus dietary and lifestyle advice versus placebo plus the same advice	Birthweight > 4000 g occurred in 16% versus 14% (adjusted RR 0.97, 95% CI 0.65–1.47). Weekly gestational weight gain was modestly lower with metformin, but clinically important pregnancy and birth outcomes were not improved.	The primary fetal outcome was negative. Metformin increased the probability of gestational weight gain below recommendations. The intervention addressed overweight or obesity without diabetes, limiting extrapolation to GDM or T2DM.

**Table 4:** Principal human studies of long-term offspring outcomes after prenatal metformin exposure and major sources of heterogeneity.

Study and Setting	Maternal Indication and Comparison	Offspring Age and Follow-Up	Outcomes Assessed	Principal Findings	Major Limitations and Sources of Heterogeneity
MiG TOFU; Adelaide and Auckland, Australia and New Zealand	GDM; metformin with supplemental insulin when required versus insulin	Adelaide: 7 years, 109/181 eligible children (60%); Auckland: 9 years, 99/396 (25%)	Anthropometry, skinfolds, bioimpedance, DXA, abdominal MRI, fasting glucose, insulin, lipids, liver markers, leptin, and adiponectin	No between-group differences at 7 years in Adelaide. At 9 years in Auckland, metformin-exposed children were larger according to weight, waist and arm circumferences, but total and abdominal body-fat percentages and metabolic biomarkers were similar.	Markedly different follow-up rates between centers; low Auckland participation; differences in maternal BMI, treatment glycemia, and birth outcomes between sites; metformin group included supplemental insulin; multiple anthropometric outcomes.
Finnish follow-up of two randomized GDM trials	GDM; metformin versus insulin	9 years; 172 children, corresponding to 55% of the original cohort	Anthropometry, blood pressure, OGTT, lipoproteins, and apolipoproteins	BMI, waist-to-height ratio, and glucose metabolism were similar. Metformin-exposed children had higher HDL cholesterol and lower LDL cholesterol and apolipoprotein B; selected differences were observed primarily in boys.	Two open-label parent trials; 45% attrition; Finnish population; multiple secondary and sex-stratified comparisons; limited power for uncommon metabolic outcomes.
Born in Bradford cohort, United Kingdom	GDM; metformin versus insulin, with a non-GDM reference group	Birth to 60 months; 76 metformin-exposed, 420 insulin-exposed, and 9171 non-GDM offspring	Longitudinal weight, height, and BMI Z score trajectories derived from routinely collected measurements	Growth trajectories were comparable after metformin and insulin exposure. By 60 months, mean BMI Z scores were also comparable with the non-GDM reference group.	Observational treatment allocation; small metformin group; residual confounding by treatment indication and severity; routine clinical measurements; no detailed body-composition or biochemical assessment.
MiTy Kids; Canada and Australia	Pregestational T2DM; metformin plus insulin versus placebo plus insulin	3–24 months; 283/465 eligible children (61%)	Serial weight, length or height, BMI Z score, and skinfold thickness	At 24 months, BMI Z score and the sum of skinfolds were similar, with no overall difference in BMI trajectory. A different trajectory in boys was exploratory.	Follow-up limited to two years; 39% non-participation; insufficient power for sex-specific outcomes; no evaluation of later cardiometabolic or pubertal outcomes.
Follow-up of the Pilot and PregMet PCOS trials, Norway	PCOS; metformin 1700–2000 mg/day versus placebo from the first trimester until delivery	Up to 4 years; 182 children from two randomized trials	Weight, height, BMI Z score, head circumference, and overweight or obesity	At four years, metformin-exposed children had higher weight and BMI Z scores. Overweight or obesity occurred in 32% versus 18% (OR 2.17, 95% CI 1.04–4.61).	Combination of two parent trials; predominantly Norwegian population; comparison with placebo rather than insulin; multiple growth assessments; postnatal lifestyle and familial factors incompletely characterized.
PedMet follow-up of PregMet, Norway	PCOS; metformin 2000 mg/day versus placebo	5–10 years; 141/255 invited children (55%)	BMI Z score, anthropometry, body composition, and selected cardiometabolic markers	Metformin-exposed children had a higher mean BMI Z score by 0.41 (95% CI 0.03–0.78). Evidence for consistent adverse metabolic biomarker differences was limited.	Approximately 45% attrition; broad age range at assessment; limited ethnic and geographic diversity; single maternal indication; insufficient evidence after puberty.
GRoW childhood follow-up, Australia	Maternal overweight or obesity without diabetes; metformin plus dietary and lifestyle intervention versus placebo plus the same intervention	6 months, 18 months, and 3–5 years; children from the 513 eligible trial births, with participation varying by assessment	Weight, height, BMI Z score, anthropometry, diet, physical activity, and neurodevelopment	The proportion with BMI Z score above the 85th percentile was similar between groups at all time points. No childhood benefit of prenatal metformin was demonstrated.	Participation varied across follow-up periods; both groups received lifestyle intervention; high childhood overweight prevalence in both groups; maternal obesity and postnatal environment likely remained dominant determinants.
Finnish nationwide register-based cohort	Mixed maternal indications excluding type 1 diabetes; metformin only, insulin only, or combined treatment	Childhood follow-up through national healthcare registers; 3967 metformin-only, 5273 insulin-only, and 889 combination-exposed children	Diagnosed obesity, glucose disturbances, diabetes, hypertension, PCOS, and motor-social developmental problems	Metformin was not associated with increased long-term diagnosed outcomes compared with insulin. An increased SGA signal was identified at birth, while adverse findings with combined therapy may have reflected greater disease severity.	Non-randomized treatment selection; mixed maternal indications; residual confounding by indication; limited data on glycemic control, BMI, adherence, placental function, breastfeeding, and lifestyle; register diagnoses may miss subclinical phenotypes.

**Table 5:** Indication-specific balance of maternal benefits, fetal and neonatal outcomes, and long-term uncertainties associated with metformin use during pregnancy.

Maternal Indication	Principal Maternal Benefits	Fetal and Neonatal Benefits	Potential Fetal or Neonatal Risks and Uncertainties	Long-Term Offspring Evidence	Overall Narrative Confidence and Clinical Interpretation
Gestational diabetes mellitus	Comparable glycemic control to insulin in selected women; generally lower gestational weight gain; lower maternal hypoglycemia risk; oral administration and greater treatment acceptability. Supplemental insulin remains necessary in a substantial proportion of women.	Lower risk of neonatal hypoglycemia in several randomized trials and meta-analyses; modestly lower mean birthweight; reduced macrosomia or large-for-gestational-age birth in some comparisons.	Placental transfer produces direct fetal exposure. Lower birthweight should not automatically be interpreted as beneficial. Available evidence does not demonstrate a consistent increase in SGA, but results vary according to comparator, diagnostic criteria, treatment timing, dose, and supplemental insulin use.	Most studies have not identified consistent differences in childhood body-fat percentage, glucose metabolism, blood pressure, or neurodevelopment. Transient differences in infant growth and larger anthropometric measures in individual MiG TOFU cohorts remain difficult to interpret because of attrition and geographic heterogeneity. Adult outcomes are unavailable.	Moderate-to-high confidence for selected short-term maternal and neonatal effects; low confidence for long-term developmental outcomes. Metformin may be considered in selected patients but should not delay insulin when glycemic targets are not achieved.
Pregestational type 2 diabetes mellitus	When added to insulin, metformin may improve glycemic control, reduce insulin requirements, limit gestational weight gain, and reduce cesarean delivery in some studies.	Lower mean birthweight, fewer very large infants, reduced neonatal adiposity, and lower risk of extreme fetal overgrowth.	MiTy identified an increased SGA rate, particularly relevant in women with chronic hypertension, nephropathy, or potentially impaired placental reserve. Reduction in LGA and increase in SGA should be interpreted as a potential fetal growth trade-off rather than as independent outcomes. Broad composite neonatal morbidity was not reduced in MiTy or MOMPOD.	MiTy Kids found no overall difference in BMI Z score or skinfold thickness at 24 months, but follow-up was incomplete and too short to evaluate later insulin resistance, adiposity, pubertal development, or cardiovascular health.	Moderate confidence for reductions in insulin requirement, maternal weight gain, and fetal overgrowth; moderate confidence for the T2DM-specific SGA signal; low confidence for long-term offspring outcomes. Routine addition of metformin to insulin is not supported for all women with T2DM.
Polycystic ovary syndrome	Possible reduction in late miscarriage or preterm birth, although the primary PregMet2 composite outcome was not statistically significant. Metformin does not reliably prevent GDM.	No consistent reduction in major neonatal morbidity. Some studies reported larger neonatal head circumference, but the clinical relevance is uncertain.	Routine continuation solely to prevent GDM is not supported. The balance between possible obstetric benefit and prolonged fetal exposure remains uncertain. Maternal PCOS phenotype, hyperandrogenism, infertility treatment, obesity, and insulin resistance may independently influence offspring outcomes.	PregMet and PedMet follow-up studies reported higher childhood BMI or overweight prevalence after prenatal metformin exposure. These findings were derived mainly from Norwegian cohorts with substantial attrition and may reflect an interaction between treatment, maternal PCOS, familial susceptibility, and postnatal exposures.	Low-to-moderate confidence for a possible reduction in preterm birth; moderate confidence that metformin does not prevent GDM; low confidence regarding childhood growth and other long-term outcomes. Preconception use should not lead automatically to continuation until delivery.
Maternal overweight or obesity without diabetes	Modest reduction in gestational weight gain in several trials. Some individual studies reported lower pre-eclampsia rates, but this finding has not been consistent across trials.	No consistent improvement in birthweight, macrosomia, neonatal morbidity, or other major perinatal outcomes.	Direct fetal exposure occurs in the absence of an established hyperglycemic treatment indication. Insufficient maternal weight gain may occur, and reduction in maternal weight gain has not been shown to improve fetal or neonatal health consistently.	GRoW childhood follow-up did not demonstrate a reduction in childhood overweight or obesity. Available follow-up remains incomplete and is strongly influenced by maternal obesity and the shared postnatal environment.	Moderate confidence that gestational weight gain may be modestly reduced; moderate confidence that clinically important fetal or childhood benefits have not been demonstrated. Maternal obesity alone is not an established indication for routine metformin treatment during pregnancy.

**Table 6:** Side-by-side interpretation of apparently conflicting findings regarding fetal growth and long-term offspring outcomes after prenatal metformin exposure.

Evidence Source	Maternal Indication/Population	Comparator	Geographic Setting	Follow-Up/Outcome Definition	Follow-Up Completeness/Sample	Control of Confounding/Methodological Considerations	Main Finding and Interpretation
Tarry-Adkins et al., 2019 [36]	GDM only	Metformin vs. insulin	Multinational; contributing studies from Europe, the Americas, Asia, Australia, and Africa	Infant weight at 18–24 months; BMI and other growth measures at 5–9 years	411 infants from 2 studies at 18–24 months; 520 children from 3 studies at 5–9 years; childhood evidence derived from a small subset of the total evidence base	Restricted to randomized or prospective randomized GDM comparisons, but heterogeneous GDM diagnostic criteria, metformin dosing, treatment protocols, and limited childhood follow-up	Metformin-exposed infants were heavier at 18–24 months and had higher BMI at 5–9 years despite lower birthweight. The finding suggested possible age-specific catch-up growth but was based on relatively few children.
Fu et al., 2025 [54]	Multiple insulin-resistant maternal conditions, including diabetes, PCOS, and obesity	Metformin-exposed vs. non-exposed or alternative-treatment groups; comparator varied by study	Multinational evidence synthesis	BMI and adiposity outcomes; analyses stratified at 1–3, 3–6, and 6–11 years; weighted mean age at oldest follow-up approximately 4.4 years	18 studies; 7975 metformin-exposed children and more than 1 million non-exposed comparators	Included RCT follow-up and observational evidence; study-level risk of bias was assessed and certainty evaluated using GRADE; residual confounding and comparator heterogeneity remained	A small BMI increase was observed at 1–3 years, but no overall BMI difference persisted at 3–6 or 6–11 years. The broader evidence base therefore does not confirm a uniform persistent increase in childhood adiposity.
PregMet/PedMet follow-up [46,47]	PCOS	Metformin vs. placebo from early pregnancy	Norway	BMI, weight, and overweight/obesity at 4 years; BMI Z score and cardiometabolic outcomes at 5–10 years	182 children in the 4-year follow-up; 141 of 255 invited children (55%) participated in the 5–10-year PedMet follow-up	Randomized parent trials reduce treatment-selection confounding, but offspring follow-up was incomplete, cohorts were geographically concentrated, and PCOS-related metabolic and hormonal factors remain specific to this indication	Higher childhood BMI and overweight prevalence were observed after metformin exposure. The persistence of this signal in PCOS but not consistently in diabetes or maternal obesity suggests indication-specific effect modification rather than a universal metformin effect.
MiTy and MiTy SGA analysis [40,42]	Pregestational T2DM; higher baseline metabolic, vascular, and placental risk	Metformin plus insulin vs. placebo plus insulin	Canada and Australia	SGA at birth; SGA defined according to gestational-age-adjusted birthweight criteria	502 women were randomized in MiTy; 460 infants were eligible for the secondary SGA predictor analysis	Randomized treatment allocation; secondary multivariable analysis evaluated maternal predictors including chronic hypertension and nephropathy	SGA occurred more frequently with metformin. Both metformin exposure and chronic hypertension and/or nephropathy independently predicted SGA, and risk was greatest when these factors coexisted. This suggests interaction with the maternal vascular–placental phenotype.
Finnish register study: metformin vs. insulin [39]	Mixed diabetes indications excluding T1DM	Metformin vs. insulin	Finland	SGA and other birth outcomes; long-term metabolic and developmental diagnoses	Large population-based register cohort with several thousand metformin- and insulin-exposed pregnancies	Inverse probability of treatment weighting was used, but residual confounding by disease severity and treatment indication remains possible	Metformin was associated with higher SGA risk when insulin was the comparator. Because insulin-treated women may differ substantially in diabetes severity and comorbidity, the association cannot be attributed confidently to metformin alone.
Finnish register study: metformin vs. non-pharmacologically treated GDM [53]	GDM	Metformin vs. non-pharmacological management	Finland	SGA and LGA at birth	Large population-based cohort; additional analyses restricted to maternal GDM	Inverse probability weighting and restriction to GDM reduced, but could not eliminate, residual confounding by disease severity and indication for pharmacotherapy	No increased SGA risk was observed when the comparator was non-pharmacologically treated GDM. The contrast with the insulin-referenced analysis demonstrates how comparator selection materially alters the observed association.

Abbreviations: BMI, body mass index; GDM, gestational diabetes mellitus; PCOS, polycystic ovary syndrome; SGA, small for gestational age; T2DM, type 2 diabetes mellitus. Apparent discordance among studies should be interpreted in relation to maternal indication, disease severity, comparator treatment, gestational timing and duration of exposure, vascular and placental comorbidity, offspring age, attrition, and postnatal confounding.

## Data Availability

No new data were created or analyzed in this study. Data sharing is not applicable to this article.

## Supplementary Materials

The following supporting information can be downloaded at: https://www.mdpi.com/article/10.3390/biomedicines14081867/s1, Table S1. Study-level evidence map of the 62 publications contributing to the narrative synthesis.

## Abbreviations

AMPK, AMP-activated protein kinase; BMI, body mass index; CI, confidence interval; DOHaD, Developmental Origins of Health and Disease; FGR, fetal growth restriction; GDM, gestational diabetes mellitus; IPTW, inverse probability of treatment weighting; LGA, large for gestational age; MATE, multidrug and toxin extrusion protein; mTOR, mechanistic target of rapamycin; OCT, organic cation transporter; OR, odds ratio; PCOS, polycystic ovary syndrome; PGC-1α, peroxisome proliferator-activated receptor gamma coactivator 1-alpha; RCT, randomized controlled trial; RR, relative risk; SGA, small for gestational age; T2DM, type 2 diabetes mellitus; WHO, World Health Organization.
