# Supplementary material for: Metformin Exposure in Pregnancy and Fetal Programming: A Focused Review of Contemporary Evidence
Source: Biomedicines. 2026 Aug 20;14(8):1867. doi: 10.3390/biomedicines14081867 (PMC13509759; doi:10.3390/biomedicines14081867)
Supplement: Supplementary file 1 [file biomedicines-14-01867-s001.zip › biomedicines-4417357-supplementary.pdf]

Supplementary Materials

Supplementary Table S1. Study-level evidence map of publications contributing to the narrative synthesis

Reference numbers correspond to the final numbering in the main manuscript. The table includes 62 publications and maps their role, design, population or experimental model, principal contribution, limitations, and domain-specific narrative confidence. Confidence descriptors are GRADE-informed narrative judgments, as defined in Section 2.6 of the manuscript, and do not represent formal outcome-specific GRADE ratings or de novo study-level risk-of-bias assessments.

A. Foundational, methodological, and guideline sources (References 1–15)

Ref.	Publication	Design and population/model	Exposure, comparator, or follow-up	Principal contribution to the review	Main limitations	Narrative confidence
1	Barker, 1990 — The fetal and infant origins of adult disease	Conceptual epidemiological paper; fetal-origins framework.	Historical observations linking early-life growth with later disease.	Provided the conceptual foundation for developmental programming and the later DOHaD framework.	Not metformin-specific; hypothesis-generating historical evidence rather than contemporary causal evidence.	Contextual; not graded
2	Gluckman et al., 2008 — Effect of in utero and early-life conditions on adult health and disease	Authoritative narrative review of developmental plasticity and DOHaD.	Human and experimental evidence across early-life exposures.	Extended the fetal-origins hypothesis to life-course developmental adaptation and later cardiometabolic disease.	Broad conceptual synthesis; not specific to diabetes, pregnancy pharmacotherapy, or metformin.	Contextual; not graded
3	Fleming et al., 2018 — Origins of lifetime health around the time of conception	Lancet review/series article focused on periconception health.	Preconception and early embryonic exposures.	Supported the importance of the preconception and first-trimester windows in developmental programming.	Broad, multidisciplinary review; no direct assessment of metformin exposure.	Contextual; not graded
4	Goyal et al., 2019 — Epigenetic responses and the developmental origins of health and disease	Narrative mechanistic review.	Epigenetic pathways linking early-life exposures with later disease.	Provided a framework for interpreting DNA methylation, histone modification, and transcriptional regulation.	Mechanistic and cross-disciplinary; limited direct evidence from metformin-exposed human offspring.	Contextual; not graded
5	Prouillac and Lecoeur, 2010 — Placental exposure to xenobiotics and membrane transporters	Narrative review of placental transport and experimental models.	Placental membrane transporters, perfusion models, and fetal xenobiotic exposure.	Supported the distinction between maternal pharmacokinetics, placental transfer, and fetal exposure.	Not metformin-specific; transporter knowledge has evolved since publication.	Contextual; moderate relevance
6	Ornoy et al., 2021 — Diabetes during pregnancy and long-term offspring effects	Narrative review.	Maternal diabetes, placental dysfunction, congenital anomalies, and long-term offspring health.	Supported interpretation of maternal disease as an independent developmental exposure and confounder.	Broad review combining diabetes types and outcomes; not designed to isolate metformin effects.	Contextual; moderate relevance
7	Rena et al., 2017 — Mechanisms of action of metformin	Mechanistic review.	Hepatic glucose production, mitochondrial function, AMPK-related and AMPK-independent pathways.	Provided the general pharmacological framework for maternal metabolic and cellular effects of metformin.	Predominantly non-pregnant evidence; direct placental and fetal applicability is limited.	Contextual; moderate relevance
8	Eyal et al., 2010 — Pharmacokinetics of metformin during pregnancy	Prospective pregnancy pharmacokinetic study with maternal, cord, and lactation sampling.	Oral metformin during pregnancy and postpartum; fetal and breast-milk exposure.	Demonstrated increased renal clearance in pregnancy, substantial fetal exposure at delivery, and very low infant exposure through milk.	Small pharmacokinetic sample; heterogeneous indications and dosing; cord concentrations represent one time point.	Moderate
9	McEvoy et al., 2025 — Narrative review of metformin in pregnancy	Contemporary narrative review across maternal indications.	GDM, pregestational diabetes, obesity, emerging indications, and short-term outcomes.	Provided a current clinical overview and highlighted indication-specific benefit–risk interpretation.	Narrative design; limited emphasis on formal developmental-programming integration and long-term evidence mapping.	Contextual; high relevance
10	Mayadunne et al., 2025 — Long-term effects of metformin on offspring health	Contemporary narrative review.	Growth, adiposity, metabolic and other long-term outcomes after prenatal exposure.	Supported the identification of persistent evidence gaps beyond childhood and the need for indication-specific interpretation.	Underlying evidence is heterogeneous, with small follow-up cohorts and limited data after puberty.	Contextual; high relevance
11	Sukhera, 2022 — Narrative reviews: Flexible, rigorous, and practical	Methodological commentary.	Planning, transparency, scope, and synthesis in narrative reviews.	Supported the methodological rationale for a focused narrative review spanning heterogeneous evidence domains.	General methodological guidance; not a validated study-quality instrument.	Methodological; not graded
12	Baethge et al., 2019 — SANRA scale	Development and validation of a narrative-review quality-assessment scale.	Six SANRA domains for narrative-review reporting and quality.	Informed transparent reporting of the review question, search, referencing, evidence level, and endpoints.	SANRA assesses review quality rather than individual-study risk of bias or outcome-specific certainty.	Methodological; not graded
13	American Diabetes Association, 2026 — Management of diabetes in pregnancy	Annual clinical practice guideline.	GDM and preexisting diabetes management, glycemic targets, and pharmacological treatment.	Established insulin as preferred therapy in major guidance and contextualized selected use of metformin.	Guideline recommendations reflect evidence interpretation, regulatory context, and feasibility; not primary evidence.	Guideline; certainty varies

Ref.	Publication	Design and population/model	Exposure, comparator, or follow-up	Principal contribution to the review	Main limitations	Narrative confidence
**14**	Wyckoff et al., 2025 — Endocrine Society/ESE guideline for preexisting diabetes	Joint evidence-based clinical practice guideline using GRADE.	Preconception, pregnancy, delivery, and postpartum care in preexisting diabetes.	Provided the recommendation against routine addition of metformin to insulin in T2DM because of the LGA–SGA trade-off and childhood uncertainty.	Several recommendations were based on low or very-low certainty evidence.	Guideline; low certainty for metformin recommendation
**15**	Zhang et al., 2019 — GRADE approach in guideline development	Methodological article.	GRADE domains, certainty judgments, and use of systematic reviews in recommendations.	Informed the structured, GRADE-informed narrative confidence descriptors used in the review.	The manuscript did not perform formal outcome-specific GRADE profiles.	Methodological; not graded

B. Pharmacokinetics, placental transport, mechanistic, and animal evidence (References 16–32)

Ref.	Publication	Design and population/model	Exposure, comparator, or follow-up	Principal contribution to the review	Main limitations	Narrative confidence
**16**	Liao et al., 2020 — Effects of pregnancy on metformin pharmacokinetics	Human longitudinal pharmacokinetic study.	500- and 1000-mg dosing during pregnancy and comparison with non-pregnant/postpartum exposure.	Showed increased oral and renal clearance, altered volume of distribution, dose-dependent bioavailability, and variable maternal–fetal exposure.	Small pharmacokinetic cohort; no long-term clinical outcomes; exposure at delivery does not measure cumulative fetal dose.	Moderate
**17**	Bergagnini-Kolev et al., 2017 — Renal cation-transporter activity in pregnancy	Human pharmacokinetic study using an endogenous probe.	Pregnant patients prescribed metformin; renal secretion of N1-methylnicotinamide.	Supported increased renal cation secretion during pregnancy and the role of OCT2/MATE pathways in maternal clearance.	Indirect transporter probe; small study; does not quantify placental or fetal transporter activity.	Moderate-to-low
**18**	Lee et al., 2018 — OCT3 facilitates fetal metformin exposure	Human placental localization plus pregnant-mouse transporter study.	OCT3 expression and Oct3 knockout during maternal metformin exposure.	Localized OCT3 to the fetal-facing placental membrane and showed approximately 47% lower fetal exposure in knockout mice.	Key functional estimate was derived from mice; contribution to individual human fetal exposure remains unquantified.	Moderate mechanistic
**19**	Anoshchenko et al., 2020 — Gestational age-dependent placental transporter abundance	Quantitative proteomic study of human placental tissue.	Placental transporter abundance from early pregnancy to term.	Showed that OCT3 abundance increases with gestational age, supporting timing-dependent fetal disposition.	Protein abundance does not prove transport function; sample availability and gestational-stage representation were limited.	Moderate-to-low mechanistic
**20**	Suzuki et al., 2025 — Rat-specific placental MATE1	Comparative experimental transporter study.	Rat and human placental transporter expression and fetal transfer.	Identified species-specific placental MATE1 as a potential explanation for lower fetal metformin exposure in rats.	Animal and comparative mechanistic evidence; direct human clinical relevance is inferential.	Low/exploratory
**21**	Lofthouse et al., 2023 — Modelling metformin transfer in perfused human placenta	Ex vivo human placental perfusion plus computational modelling.	Paracellular diffusion and OCT3-mediated transfer across term placenta.	Supported bidirectional transfer involving both paracellular and transporter-mediated pathways.	Term placental model; assumptions are model-dependent; does not represent early gestation or chronic exposure.	Moderate-to-low mechanistic
**22**	Abolhassani et al., 2023 — Major malformations after early exposure	Systematic review and meta-analysis of 11 studies.	First-trimester metformin exposure in PCOS or pregestational diabetes versus unexposed/comparator pregnancies.	Did not identify a meaningful overall increase in major congenital malformations.	Small randomized samples, observational confounding, heterogeneity by indication, and imprecise confidence intervals.	Moderate
**23**	Tarry-Adkins et al., 2022 — Human placental energy production and oxidative stress	Primary human trophoblast experiment.	Clinically relevant metformin concentrations in trophoblasts from term placentas.	Reduced mitochondrial respiration and ATP production while also reducing oxidative-stress markers.	Isolated term trophoblasts; short experimental exposure; net in vivo clinical effect cannot be determined.	Low mechanistic
**24**	Hebert and Myatt, 2023 — Sexually dimorphic syncytiotrophoblast responses	Human syncytiotrophoblast experiment.	Cells from lean, obese, and GDM pregnancies with male and female fetuses; concentration-response exposure.	Demonstrated concentration-, maternal-phenotype-, and fetal-sex-dependent mitochondrial and oxidative responses.	Many pronounced effects occurred at 1–10 mmol/L, above typical maternal therapeutic concentrations; term cells only.	Low/exploratory
**25**	Grace et al., 2019 — Placental mTOR signaling in mice	Pregnant-mouse experimental study.	High-fat diet with metformin intervention.	Supported biological plausibility that metformin can modify placental AMPK–mTOR-related nutrient-sensing pathways.	Animal model; pathway changes do not establish clinical benefit or harm in human offspring.	Low/exploratory
**26**	Jiang et al., 2020 — Epigenetic regulation of placental mitochondrial biogenesis	Human placental explants with complementary mouse experiments.	Diabetic placenta, metformin exposure, AMPK/PGC-1α, histone acetylation, and promoter methylation.	Suggested sex-dependent epigenetic and mitochondrial-biogenesis responses, particularly in male placental tissue.	Small term-placenta sample; mixed human and animal evidence; persistence in offspring was not assessed.	Low/exploratory
**27**	Hattersley et al., 2025 — Placental ageing in humans and mice	Randomized human placental substudy with complementary experimental models.	Placental samples from 105 women with obesity randomized to metformin or placebo.	Found no differences in placental gene expression, DNA methylation, telomere length, fibrosis, calcification, or ageing markers.	Obesity-specific population; placental endpoints do not exclude effects in other indications or later offspring outcomes.	Moderate
**28**	Newman et al., 2026 — Fetal sex in the EMERGE trial	Secondary analysis of a randomized GDM trial.	Metformin versus placebo, stratified by fetal sex.	Exploratory findings suggested greater maternal glycemic effects in male pregnancies and more birthweight below 2500 g among metformin-exposed females.	Sex-by-treatment interactions were not statistically significant; secondary, underpowered, multiple-comparison analysis.	Low/exploratory
**29**	Schoonejans et al., 2022 — Sex-specific aged mouse-offspring outcomes	Mouse developmental-programming study.	Metformin during obese glucose-intolerant pregnancy with later-life offspring assessment.	Reported sex- and age-dependent differences in adiposity, inflammation, hepatic lipid, leptin, and insulin phenotypes.	Animal model; findings depended on maternal phenotype and postnatal environment; not directly transferable to humans.	Low/exploratory
**30**	van Hoorn et al., 2024 — Animal offspring systematic review and meta-analysis	Systematic review/meta-analysis of 37 animal studies; 3133 offspring from 716 litters.	Prenatal metformin exposure and anthropometric, cardiovascular, and metabolic offspring outcomes.	Found no significant overall effect across pooled animal outcomes.	Very high heterogeneity, mostly rodent models, poor methodological reporting, and frequently unclear risk of bias.	Low

Ref.	Publication	Design and population/model	Exposure, comparator, or follow-up	Principal contribution to the review	Main limitations	Narrative confidence
**31**	Salomäki et al., 2013 — Prenatal metformin and adult high-fat-diet response	Mouse developmental study.	Prenatal exposure in metabolically healthy dams; adult offspring challenged with high-fat diet.	Reported greater weight gain, mesenteric fat, and impaired glucose tolerance in exposed male offspring under dietary challenge.	Single animal model; sex- and diet-dependent phenotype; limited relevance to human therapeutic exposure.	Low/exploratory
**32**	Salomäki et al., 2014 — Prenatal metformin in a maternal high-fat-diet model	Mouse developmental and transcriptomic study.	Maternal high-fat diet with prenatal metformin; adult offspring metabolic challenge.	Reported altered transcriptomic responses and comparatively lower weight/adiposity with preserved glucose tolerance in the studied model.	Animal findings differ from other models and depend on maternal metabolic state and postnatal diet.	Low/exploratory

C. Gestational diabetes mellitus and GDM offspring evidence (References 33–39)

Ref.	Publication	Design and population/model	Exposure, comparator, or follow-up	Principal contribution to the review	Main limitations	Narrative confidence
**33**	Rowan et al., 2008 — MiG trial	Multicenter open-label RCT; 751 women with GDM requiring pharmacotherapy.	Metformin with supplemental insulin when required versus insulin; treatment mainly in later pregnancy.	Composite neonatal outcome was similar (32.0% vs. 32.2%); 46.3% required supplemental insulin; treatment acceptability favored metformin.	Open-label; metformin arm was frequently combination therapy; powered for short-term neonatal morbidity, not long-term development.	High-to-moderate
**34**	Rademaker et al., 2025 — SUGAR-DIP trial	Multicenter open-label noninferiority RCT; 820 women with GDM.	Sequential metformin, then glyburide and insulin if required, versus insulin.	Oral strategy did not meet noninferiority for LGA (23.9% vs. 19.9%); 79% avoided insulin.	Not a metformin-monotherapy trial; treatment-strategy comparison and open-label design limit drug-specific attribution.	High for strategy; moderate for metformin-specific inference
**35**	Brinkmann et al., 2026 — GDM safety meta-analysis	Systematic review/meta-analysis of 10 RCTs and available follow-up studies.	Metformin-based treatment versus insulin/comparator in GDM.	Lower neonatal hypoglycemia (OR 0.65) and modestly lower birthweight; no established long-term metabolic difference.	Heterogeneous diagnostic criteria, treatment protocols, supplemental insulin, and sparse long-term follow-up.	Moderate-to-high short term; low long term
**36**	Tarry-Adkins et al., 2019 — Growth after metformin versus insulin	Systematic review and meta-analysis of neonatal and childhood growth studies.	GDM treated with metformin versus insulin; infancy and childhood follow-up.	Lower birthweight, greater weight at 18–24 months, and higher BMI in mid-childhood were reported.	Few follow-up cohorts, high heterogeneity, attrition, and limited ability to separate maternal and postnatal confounding.	Moderate neonatal; low childhood
**37**	Rowan et al., 2011 — MiG TOFU at 2 years	Follow-up of the MiG RCT.	Prenatal metformin-based strategy versus insulin; offspring assessed at 2 years.	Larger mid-upper arm circumference and selected skinfolds, but similar total fat mass and body-fat percentage.	Subset follow-up, young age, multiple anthropometric outcomes, and no later cardiometabolic assessment.	Low-to-moderate
**38**	Rowan et al., 2018 — MiG TOFU at 7–9 years	Two-center follow-up of the MiG RCT; 109 Adelaide and 99 Auckland children.	Prenatal metformin-based strategy versus insulin; anthropometry, body composition, and metabolic biomarkers.	No clear differences at 7 years; Auckland children were larger at 9 years, but body-fat percentages and metabolic biomarkers were similar.	Marked center-specific attrition, low Auckland participation, geographic heterogeneity, and multiple outcomes.	Low
**39**	Brand et al., 2022 — Finnish long-term register cohort	Nationwide register-based cohort; metformin only, insulin only, and combination treatment.	Mixed maternal indications; childhood diagnoses and birth outcomes.	No increased diagnosed childhood obesity, glucose disturbance, diabetes, hypertension, PCOS, or motor-social problems versus insulin; SGA signal at birth.	Non-randomized treatment selection, mixed indications, residual confounding, limited glycemic and lifestyle data, and reliance on diagnostic codes.	Moderate observational

D. Pregestational type 2 diabetes mellitus evidence (References 40–44)

Ref.	Publication	Design and population/model	Exposure, comparator, or follow-up	Principal contribution to the review	Main limitations	Narrative confidence
**40**	Feig et al., 2020 — MiTy trial	International double-blind placebo-controlled RCT; 502 women with T2DM receiving insulin.	Metformin 1000 mg twice daily plus insulin versus placebo plus insulin from 6–22+6 weeks.	No difference in the 40% composite neonatal outcome; lower HbA1c, insulin dose, weight gain, cesarean birth, birthweight, and extreme LGA; higher SGA (13% vs. 7%).	Adjunct-to-insulin design; fetal-growth trade-off; findings are specific to established T2DM with higher vascular and placental risk.	High-to-moderate
**41**	Gordijn et al., 2016 — Consensus definition of fetal growth restriction	International Delphi consensus.	Diagnostic framework distinguishing FGR from constitutionally small fetuses and birthweight-based SGA.	Supported separate interpretation of SGA and antenatally diagnosed FGR in metformin studies.	Not a metformin study; consensus criteria require application within clinical and Doppler context.	Contextual; high relevance
**42**	Feig et al., 2022 — MiTy SGA secondary analysis	Secondary analysis of 460 eligible MiTy infants.	Predictors of SGA after metformin or placebo added to insulin.	Metformin (adjusted OR 2.26) and chronic hypertension/nephropathy (OR 3.05) predicted SGA; SGA frequency with both metformin and comorbidity was 25.0%.	Secondary analysis with relatively few SGA events; cannot fully separate metformin effect from vascular and placental disease.	Moderate
**43**	Boggess et al., 2023 — MOMPOD trial	Multicenter double-blind RCT; 831 randomized and 794 analyzed.	Metformin 1000 mg twice daily plus insulin versus placebo plus insulin in preexisting T2DM or diabetes diagnosed before 23 weeks.	No significant reduction in composite neonatal morbidity (71% vs. 74%); lower odds of LGA (adjusted OR 0.63).	Combined preexisting and early-diagnosed diabetes; stopped at 75% accrual for futility; LGA reduction was a component-level finding.	High-to-moderate
**44**	Feig et al., 2023 — MiTy Kids	Prospective follow-up of the MiTy RCT; 283/465 eligible children (61%).	Prenatal metformin plus insulin versus placebo plus insulin; 3–24 months.	Similar BMI Z score, skinfold thickness, and overall BMI trajectory at 24 months; sex-specific trajectory in boys was exploratory.	39% non-participation, follow-up limited to 2 years, and insufficient power for sex-specific or later cardiometabolic outcomes.	Low-to-moderate

E. Polycystic ovary syndrome evidence (References 45–47)

Ref.	Publication	Design and population/model	Exposure, comparator, or follow-up	Principal contribution to the review	Main limitations	Narrative confidence
**45**	Løvvik et al., 2019 — PregMet2 trial	Multicenter double-blind placebo-controlled RCT; 487 pregnant women with PCOS.	Metformin up to 1000 mg twice daily from late first trimester to delivery versus placebo.	Primary late-miscarriage/preterm-birth outcome was not significant (5% vs. 10%); post hoc pooled analysis suggested benefit; GDM was not prevented.	Primary estimate was imprecise; positive pooled result was post hoc; PCOS phenotype and fertility treatment add heterogeneity.	Moderate for no GDM prevention; low-to-moderate for obstetric benefit
**46**	Hanem et al., 2018 — PCOS offspring follow-up at 4 years	Follow-up of two randomized PCOS trials; 182 children.	Metformin 1700–2000 mg/day versus placebo from first trimester to delivery.	Higher weight and BMI Z score; overweight/obesity occurred in 32% versus 18% (OR 2.17).	Combined parent trials, predominantly Norwegian population, attrition, multiple growth outcomes, and incompletely measured postnatal factors.	Low-to-moderate
**47**	Hanem et al., 2019 — PedMet follow-up	Follow-up of PregMet; 141/255 invited children (55%), aged 5–10 years.	Prenatal metformin 2000 mg/day versus placebo.	Higher mean BMI Z score by 0.41; no consistent adverse pattern across metabolic biomarkers.	Approximately 45% attrition, broad age range, limited geographic diversity, and no evidence after puberty.	Low

F. Maternal overweight or obesity without diabetes (References 48–51)

Ref.	Publication	Design and population/model	Exposure, comparator, or follow-up	Principal contribution to the review	Main limitations	Narrative confidence
**48**	Chiswick et al., 2015 — EMPOWaR trial	Multicenter double-blind placebo-controlled RCT; 449 randomized, 434 analyzed.	Metformin up to 2500 mg/day from 12–16 weeks to delivery in women with obesity and normal glucose tolerance.	Mean birthweight was virtually identical and the standardized birthweight outcome was not improved.	No hyperglycemic indication; no demonstrated fetal benefit; trial not designed for long-term developmental safety.	High for negative birthweight finding
**49**	Syngelaki et al., 2016 — Metformin in severe obesity without diabetes	Double-blind placebo-controlled RCT; 450 randomized, 400 analyzed.	Metformin 3 g/day from 12–18 weeks to delivery versus placebo.	No significant reduction in neonatal birthweight Z score; lower gestational weight gain and lower pre-eclampsia in this trial.	Pre-eclampsia was a secondary finding not consistently replicated; higher gastrointestinal adverse effects; no clear neonatal benefit.	High for birthweight; low-to-moderate for pre-eclampsia
**50**	Dodd et al., 2019 — GRoW trial	Multicenter double-blind placebo-controlled RCT; 524 randomized, 514 analyzed.	Metformin up to 2000 mg/day plus lifestyle advice versus placebo plus the same advice.	No improvement in birthweight >4000 g or major pregnancy/neonatal outcomes; modestly lower weekly gestational weight gain.	No diabetes indication, lifestyle cointervention, and increased probability of gestational weight gain below recommendations.	High for no major fetal benefit
**51**	Deussen et al., 2023 — GRoW childhood follow-up	Prospective offspring follow-up from 6 months to 3–5 years.	Prenatal metformin plus lifestyle intervention versus placebo plus the same intervention.	Similar frequency of BMI Z score above the 85th percentile and no demonstrated childhood anthropometric or neurodevelopmental benefit.	Variable participation, high overweight prevalence in both groups, and strong influence of maternal obesity and postnatal environment.	Low

G. Additional contemporary clinical, long-term, immune, endocrine, lactation, and preference evidence (References 52–62)

Ref.	Publication	Design and population/model	Exposure, comparator, or follow-up	Principal contribution to the review	Main limitations	Narrative confidence
**52**	Dunne et al., 2023 — EMERGE trial	Double-blind placebo-controlled RCT; 535 pregnancies in 510 women with GDM.	Early metformin up to 2500 mg/day plus usual care versus placebo plus usual care.	Primary composite was not significantly reduced; metformin delayed insulin initiation, improved selected glycemic measures, reduced weight gain, and produced smaller neonatal anthropometry without major morbidity differences.	Two Irish centers; favorable maternal and neonatal findings were mainly secondary; long-term offspring data are not yet available.	High-to-moderate
**53**	Brand et al., 2022 — Abnormal growth outcomes at birth	Finnish nationwide register-based cohort.	Metformin, insulin, combination therapy, and non-pharmacologically treated GDM comparators.	SGA risk differed by comparator: increased versus insulin in some analyses but not versus non-pharmacologically treated GDM, illustrating confounding by indication.	Non-randomized treatment allocation, mixed disease severity, incomplete glycemic and placental information, and residual confounding.	Moderate observational
**54**	Fu et al., 2025 — Childhood adiposity meta-analysis	Systematic review/meta-analysis of 18 studies; 7975 exposed children and >1 million comparators.	Any prenatal metformin indication; childhood adiposity at a weighted mean age of 4.4 years.	No overall BMI difference at the oldest follow-up; slightly higher BMI at 1–3 years but not later; higher BMI signal in PCOS subgroup only.	Low certainty, heterogeneous indications and comparators, several overlapping parent cohorts, and limited follow-up beyond childhood.	Moderate overall; low indication-specific
**55**	Dutta et al., 2024 — Outcomes at 5–11 years after diabetes treatment	Systematic review and meta-analysis of available follow-up cohorts.	Metformin versus insulin in pregnancies complicated by diabetes.	No significant differences in BMI, waist-to-height ratio, fat mass, body-fat percentage, visceral or liver fat, or major metabolic biomarkers.	Few cohorts, wide confidence intervals, statistical heterogeneity, attrition, and non-independent reports from shared parent trials.	Low-to-moderate
**56**	Gordon et al., 2024 — Neurodevelopmental outcomes meta-analysis	Systematic review/meta-analysis of 7 studies and 14,042 children.	Prenatal metformin exposure versus comparator; follow-up to 14 years.	No association with infant or preschool neurodevelopmental delay, motor scores, or cognitive scores.	Broad outcomes and screening instruments, heterogeneous indications, limited detailed assessment of executive function and mental health, and sparse post-pubertal data.	Moderate for no large early delay
**57**	Ryssdal et al., 2025 — Allergy and eczema after PCOS exposure	Post hoc analysis of PregMet, PregMet2, and PedMet; 145 offspring at approximately 8 years.	Prenatal metformin versus placebo in PCOS pregnancies.	Higher reported odds of allergy (OR 4.83) and eczema (OR 2.42).	Small geographically restricted sample, wide confidence intervals, multiple outcomes, post hoc design, and no independent replication.	Low/exploratory
**58**	Hanem et al., 2021 — Steroid hormones in PCOS offspring	Follow-up study; 117/255 invited children aged 5–10 years.	Maternal PCOS status and prenatal metformin versus placebo.	Maternal PCOS was associated with higher androgen measures in daughters; metformin-exposed boys had higher 11-deoxycortisol Z score in a small subgroup.	Small sex-specific samples, multiple hormonal comparisons, early pubertal age, and uncertain clinical relevance.	Low/exploratory
**59**	Hale et al., 2002 — Transfer of metformin into human milk	Human lactation pharmacokinetic study; 7 breastfeeding women.	Median maternal dose 1500 mg/day; milk and infant plasma sampling.	Mean relative infant dose was approximately 0.28%; infant plasma levels were very low or undetectable and no short-term problems were observed.	Very small sample, healthy term infants, short observation, and no long-term follow-up.	Moderate for low milk exposure
**60**	Glueck et al., 2006 — Infant development during maternal lactation treatment	Observational clinical follow-up; 61 breastfed and 50 formula-fed infants.	Mothers with PCOS continuing metformin during lactation; all infants also exposed prenatally.	Growth, motor and social development, and intercurrent illness were similar through 6 months.	Non-random feeding groups, prenatal exposure in all infants, small sample, and no uncommon or later outcomes.	Low-to-moderate
**61**	Richardson et al., 2026 — Antidiabetic medications during lactation	Systematic review of human milk-transfer evidence and guidelines.	Twenty T2DM medications considered; only five had direct human transfer data.	Confirmed sparse evidence, low metformin infant exposure, and inconsistency between clinical guidance and the direct evidence base.	Few pharmacokinetic studies, variable reporting quality, and minimal infant clinical follow-up.	Moderate for evidence gap; low for long-term safety
**62**	Venkatesh et al., 2024 — Patient priorities and treatment satisfaction	Cross-sectional study of 144 pregnant individuals with GDM requiring pharmacotherapy.	Initial metformin versus insulin decision; patient priorities, decisional comfort, and satisfaction.	Both groups reported high decisional comfort and satisfaction; metformin users more often prioritized avoiding injections and simpler dosing.	Non-random treatment choice, single integrated-care setting, possible selection and counseling effects, and no causal clinical-outcome inference.	Moderate for patient-reported preferences

Abbreviations and interpretative notes

- AMPK, AMP-activated protein kinase; BMI, body mass index; DOHaD, Developmental Origins of Health and Disease; FGR, fetal growth restriction; GDM, gestational diabetes mellitus; GRADE, Grading of Recommendations Assessment, Development and Evaluation; LGA, large for gestational age; MATE, multidrug and toxin extrusion protein; mTOR, mechanistic target of rapamycin; OCT, organic cation transporter; PCOS, polycystic ovary syndrome; PGC-1α, peroxisome proliferator-activated receptor gamma coactivator 1-alpha; RCT, randomized controlled trial; SGA, small for gestational age; T2DM, type 2 diabetes mellitus.
- Narrative confidence refers to the specific evidence contribution for which the publication was used in the review. A single publication may provide higher confidence for one outcome and lower confidence for another.
- Contextual and methodological publications were not assigned clinical certainty ratings because they informed the conceptual framework, review conduct, or guideline context rather than a metformin-specific treatment effect.
- Experimental findings were used to assess biological plausibility and generate hypotheses. They were not interpreted as proof of causal benefit or harm in human offspring.
